# PHIStruct: improving phage–host interaction prediction at low sequence similarity settings using structure-aware protein embeddings

**DOI:** 10.1093/bioinformatics/btaf016

**Published:** 2025-01-13

**Authors:** Mark Edward M Gonzales, Jennifer C Ureta, Anish M S Shrestha

**Affiliations:** Bioinformatics Lab, Advanced Research Institute for Informatics, Computing and Networking, De La Salle University, Manila 1004, Philippines; College of Computer Studies, De La Salle University, Manila 1004, Philippines; Bioinformatics Lab, Advanced Research Institute for Informatics, Computing and Networking, De La Salle University, Manila 1004, Philippines; College of Computer Studies, De La Salle University, Manila 1004, Philippines; Walter and Eliza Hall Institute of Medical Research, Melbourne, VIC 3052, Australia; Bioinformatics Lab, Advanced Research Institute for Informatics, Computing and Networking, De La Salle University, Manila 1004, Philippines; College of Computer Studies, De La Salle University, Manila 1004, Philippines

## Abstract

**Motivation:**

Recent computational approaches for predicting phage–host interaction have explored the use of sequence-only protein language models to produce embeddings of phage proteins without manual feature engineering. However, these embeddings do not directly capture protein structure information and structure-informed signals related to host specificity.

**Results:**

We present PHIStruct, a multilayer perceptron that takes in structure-aware embeddings of receptor-binding proteins, generated via the structure-aware protein language model SaProt, and then predicts the host from among the ESKAPEE genera. Compared against recent tools, PHIStruct exhibits the best balance of precision and recall, with the highest and most stable F1 score across a wide range of confidence thresholds and sequence similarity settings. The margin in performance is most pronounced when the sequence similarity between the training and test sets drops below 40%, wherein, at a relatively high-confidence threshold of above 50%, PHIStruct presents a 7%–9% increase in class-averaged F1 over machine learning tools that do not directly incorporate structure information, as well as a 5%–6% increase over BLASTp.

**Availability and implementation:**

The data and source code for our experiments and analyses are available at https://github.com/bioinfodlsu/PHIStruct.

## 1 Introduction

Described as a silent pandemic, antimicrobial resistance is among the foremost threats to public health. If left unaddressed, it is estimated that, by 2050, the number of deaths linked to antimicrobial resistance will reach 10 million annually and the global economic cost will exceed $100 trillion (Tang *et al.* 2023, Walsh *et al.* 2023). As alternatives to conventional antibiotics, phages have recently been used in phage therapy to treat bacterial infections (Górski *et al.* 2020, Hitchcock *et al.* 2023) and in phage steering to re-sensitize pathogens to antibiotics (Ashworth *et al.* 2024). The bactericidal action of phages has also been leveraged in various settings outside the clinical domain, including crop protection (Stone *et al.* 2019), oilfield reservoir souring prevention (Pannekens *et al.* 2019), and corrosion mitigation (Harada *et al.* 2018).

Identifying phage–host pairs is crucial in actualizing these applications; however, *in vitro* experiments can be time-consuming and expensive. In order to accelerate the shortlisting of candidate pairs, several *in silico* methods that capitalize on alignment-based and alignment-free techniques have been developed (Iuchi *et al.* 2023, Nie *et al.* 2024). Alignment-based approaches (Coutinho *et al.* 2021, Shang and Sun 2021, Zielezinski *et al.* 2021) focus on finding shared genomic regions between phages and putative hosts, whereas alignment-free approaches (Leite *et al.* 2018, Boeckaerts *et al.* 2021, Li *et al.* 2021, Lu *et al.* 2021, Li and Zhang 2022, Zhou *et al.* 2022) hinge on features such as sequence composition and co-abundance profiles of phages and their hosts.

Among the key determinants of phage–host specificity are receptor-binding proteins or RBPs (Dowah and Clokie 2018, Klumpp *et al.* 2023). Located at the distal end of phages, RBPs, such as tailspike proteins, adsorb to receptors found on the surface of the host bacteria. As such, these proteins have also been engineered to reprogram the target spectrum of phage-based antibacterials (Dams *et al.* 2019, Yehl *et al.* 2019, Zampara *et al.* 2020), thereby facilitating novel therapeutic applications.

In our previous work (Gonzales *et al.* 2023), we explored the use of representation learning to convert RBPs into dense vector representations (embeddings) without the need for manual feature engineering. To generate the embeddings, we explored different protein language models, such as ESM-1b (Rives *et al.* 2021), ProtT5 (Elnaggar *et al.* 2022), and SeqVec (Heinzinger *et al.* 2019). These models were pretrained in a self-supervised fashion on sequences from large-scale protein databases (Apweiler *et al.* 2004, Suzek *et al.* 2007, Steinegger and Söding 2018, Steinegger *et al.* 2019) and have been shown to capture relevant physicochemical characteristics (Heinzinger *et al.* 2019, Rives *et al.* 2021, Elnaggar *et al.* 2022, Lin *et al.* 2023). We found that this approach presents performance improvements over using manually feature-engineered sequence properties.

From a biological perspective, additional benefit may be gained by incorporating structure information, as underscored by its role in determining function and elucidating protein-protein interaction signatures, such as complementary binding surfaces and molecular forces (Ma *et al.* 2003, Planas-Iglesias *et al.* 2013). In the particular context of phage–host interaction, recent studies have investigated the structure of RBPs to obtain insights into the mechanisms underlying host recognition and binding machinery. The structural diversity of these proteins has been found to reflect the evolutionary pressure to adapt to the surface proteins of bacteria occupying the same ecological niche (Hawkins *et al.* 2022, Cambillau and Goulet 2023, Degroux *et al.* 2023).

Indeed, we found that phages infecting the same host genus have RBPs that are structurally more similar than those infecting different host genera, particularly at low sequence similarity settings. To see this, we paired RBPs targeting hosts from the ESKAPEE genera, which are among the leading causes of nosocomial infections worldwide (Venkateswaran *et al.* 2023). The pairing was done such that the sequence similarity between the RBPs in each pair is below 40%. Using US-align (Zhang *et al.* 2022), we then computed the root mean square deviation (RMSD) between their ColabFold (Mirdita *et al.* 2022)-predicted structures. Afterwards, for every genus, we constructed two groups, each with 500 randomly sampled RBP pairs. In the first group, both RBPs in each pair target the same genus of interest. In the second group, one of the RBPs in each pair targets the genus of interest, while the other RBP targets a different genus. Performing a Mann–Whitney U test showed that, for all but one genus, the difference between the distributions of the RMSD scores in these two groups is statistically significant at a *P*-value cutoff of 0.05 (Supplementary Table S7).

These point towards the utility of integrating structure information in predicting phage–host interaction. A limitation of sequence-only protein language models (i.e. those pretrained only on sequences, without explicitly involving structure data) is that they do not directly capture interactions between residues that are in close proximity when the 3D conformation is considered. This has motivated the development of structure-aware protein language models such as ProstT5 (Heinzinger *et al.* 2024), SaProt (Su *et al.* 2024), and PST (Chen *et al.* 2024), which adopt a custom vocabulary for encoding structure information (Heinzinger *et al.* 2024, Su *et al.* 2024) or augment the architecture of an existing sequence-only model in order to inject structure bias into the output embeddings (Chen *et al.* 2024). While structure-aware embeddings have been used for downstream benchmark tasks such as thermostability, metal ion binding, and protein function prediction (Chen *et al.* 2024, Heinzinger *et al.* 2024, Su *et al.* 2024), their application to computationally predicting phage–host pairs remains unexplored.

In this paper, we introduce PHIStruct, a deep-learning model for phage–host interaction prediction that uses structure-aware embeddings of receptor-binding proteins for phage–host interaction prediction. We focused our scope on hosts belonging to the ESKAPEE genera (*Enterococcus*, *Staphylococcus*, *Klebsiella*, *Acinetobacter*, *Pseudomonas*, *Enterobacter*, and *Escherichia*), which include bacteria that are known to exhibit multidrug resistance (Ayobami *et al.* 2022, Kritsotakis *et al.* 2022) and are among the priority pathogens identified by the World Health Organization (2024) and the Antimicrobial resistance surveillance in Europe 2023 - 2021 data (2023). Our contributions are as follows:

- We constructed a dataset of protein structures, computationally predicted via ColabFold (Mirdita *et al.* 2022), of 7627 nonredundant (i.e. with duplicates removed) receptor-binding proteins from 3350 phages that target hosts from the ESKAPEE genera.
- We fed the ColabFold-predicted structures to the structure-aware protein language model SaProt (Su *et al.* 2024) in order to generate embeddings of the receptor-binding proteins. We then trained a two-hidden-layer perceptron that takes in these structure-aware embeddings as input and predicts the host genus.
- We found that our model, PHIStruct, presents improvements over state-of-the-art tools that take in sequence-only protein embeddings and feature-engineered genomic and protein sequence properties, as well as BLASTp—especially as the sequence similarity between the training and test set entries decreases. Further evaluation highlights its ability to make high-confidence predictions without heavily compromising between precision and recall. When the sequence similarity drops below 40% and the confidence threshold is set to above 50%, our tool outperforms machine learning tools that do not directly integrate structure information by 7%–9% and BLASTp by 5%–6% in terms of class-averaged F1.

## 2 Materials and methods

**Figure 1.**
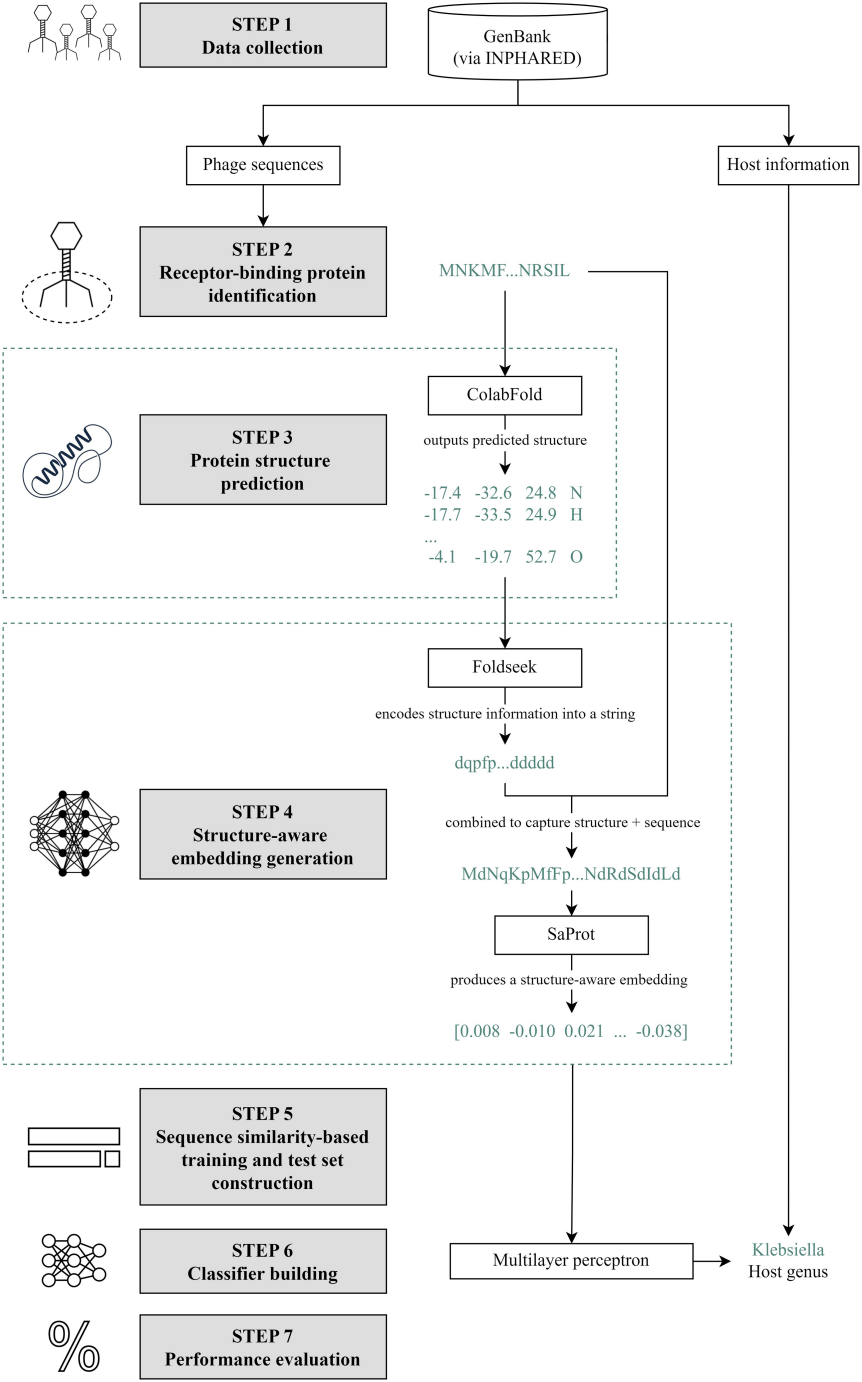
Methodology. Step 1: We collected phage genome and protein sequences from GenBank (Benson *et al.* 2007) using INPHARED (Cook *et al.* 2021). Step 2: Receptor-binding proteins (RBPs) were identified following the methodology in our previous work (Gonzales *et al.* 2023). Step 3: We fed the RBP sequences to ColabFold (Mirdita *et al.* 2022) to predict their structures. Step 4: The proteins, alongside their predicted structures, were fed to SaProt. For a protein of length *n*, the input to SaProt is $\langle \left(r_{1},f_{1}\right),\left(r_{2},f_{2}\right),\ldots ,\left(r_{n},f_{n}\right)\rangle$, where $\langle r_{1},r_{2},\ldots ,r_{n}\rangle$ is the sequence representation and $\langle f_{1},f_{2},\ldots ,f_{n}\rangle$ is the structure representation from Foldseek (van Kempen *et al.* 2024). SaProt outputs the structure-aware vector representations (embeddings). Step 5: In constructing our training and test sets, we partitioned our dataset with respect to different train-versus-test sequence similarity thresholds via CD-HIT (Fu *et al.* 2012). Step 6: We built a two-hidden-layer perceptron that takes in the SaProt embedding of an RBP as input and outputs the host genus from among the ESKAPEE genera. Step 7: We evaluated our model’s performance. Icon sources: Bacteriophage: https://static.thenounproject.com/png/1372464-200.png; Deep learning: https://static.thenounproject.com/png/2424485-200.png; Isolated icon of a neural network. concept of artificial intelligence, deep learning and machine learning: https://t4.ftcdn.net/jpg/04/30/22/13/360_F_430221349_N1HJUZArv5f4dhmzOYUzuCpxGQZ5rTO5.jpg; Percentage free icon: https://cdn-icons-png.flaticon.com/512/156/156877.png; Protein structure flat simple icon: https://t4.ftcdn.net/jpg/04/30/22/13/360_F_430221349_N1HJUZArv5f4dhmzOYUzuCpxGQZ5rTO5.jpg.

An overview of our methodology is provided in Fig. 1. The steps are described in detail in the subsequent subsections.

### 2.1 Data collection

We collected 20 941 phage genome sequences, alongside their respective protein sequences (if provided), that were uploaded to GenBank before October 2023. We used INPHARED (Cook *et al.* 2021), a pipeline for downloading phage sequences and host genus information from GenBank (Benson *et al.* 2007). For phages where INPHARED did not return a host, we retrieved the isolation host information from their respective GenBank entries, if provided. Afterwards, we discarded phages with nonbacterial hosts, leaving a total of 17 790 phages across 273 host genera.

### 2.2 Receptor-binding protein identification

Among the 17 790 collected phage sequences, 16 627 already had genome annotations from GenBank. We ran the remaining 1163 sequences without annotations through Prokka (Seemann 2014), which serves as a wrapper for a two-stage pipeline of calling Prodigal (Hyatt *et al.* 2010) for gene prediction and PHROG (Terzian *et al.* 2021), a collection of viral protein families, for functional annotation.

With all the collected sequences annotated, we identified the receptor-binding proteins (RBPs). To this end, we matched the gene product annotations against a regular expression that we proposed in our previous work (Gonzales *et al.* 2023) based on the pattern given by Boeckaerts *et al.* (2022). Since this pattern also captures RBP-related annotations that are not RBPs *per se* (e.g. tailspike adaptor proteins), we referred to the exclusion list provided by Boeckaerts *et al.* (2022) to filter out these cases.

Moreover, in view of the possibility that some proteins tagged as hypothetical might be RBPs, we converted these proteins into embeddings using the protein language model ProtBert (Elnaggar *et al.* 2022) and then passed the resulting embeddings as input to the extreme gradient boosting model PhageRBPdetect (Boeckaerts *et al.* 2022) to predict whether the proteins are RBPs.

We subsequently discarded RBPs with outlying lengths, i.e. those with lengths outside the interval $\left[Q_{1}-1.5\cdot \mathrm{IQR},Q_{3}+1.5\cdot \mathrm{IQR}\right]$, where *Q*_1_, *IQR*, and *Q*_3_ refer to the first quartile, interquartile range, and third quartile of the RBP lengths, respectively. Finally, we removed duplicate RBPs using CD-HIT (Fu *et al.* 2012), a tool for clustering protein sequences.

### 2.3 Protein structure prediction

We fed the RBP sequences to ColabFold (Mirdita *et al.* 2022), a protein structure prediction tool that accelerates the inference time of AlphaFold 2 (Jumper *et al.* 2021) by building multiple sequence alignments using MMseqs2 (Steinegger and Söding 2017) instead of JackHMMer (Johnson *et al.* 2010). ColabFold predicts the *x*-, *y*-, and *z*-coordinates of all the heavy atoms (carbon, hydrogen, nitrogen, oxygen, and sulfur) of a given protein. Supplementary Table S1 lists the parameters at which we ran ColabFold.

Our dataset consists of the predicted structures of 7627 RBPs from 3350 phages targeting hosts from the ESKAPEE genera (Table 1). The distributions of the RBP lengths and ColabFold’s confidence scores per genus are plotted in Supplementary Figs S1 and S2. We computed the confidence score for each protein by averaging the predicted local distance difference test (pLDDT) scores across its residues; the pLDDT score is a superposition-free estimate of the agreement between a predicted structure and a reference structure (Jumper *et al.* 2021). For each of the ESKAPEE genera, the mean confidence score is above 77% and the median is above 80%.

**Table 1.** Per-genus dataset characteristics.

		RBP length (a.a.)		pLDDT score	
Genus	Count	Mean	Median	Mean	Median
*Enterococcus*	170	704	620	77.57%	80.87%
*Staphylococcus*	521	510	481	82.74%	83.24%
*Klebsiella*	1535	671	658	82.00%	83.48%
*Acinetobacter*	369	599	607	77.80%	81.53%
*Pseudomonas*	1180	509	477	77.48%	79.50%
*Enterobacter*	388	545	512	81.26%	82.24%
*Escherichia*	3464	622	581	81.39%	82.42%

We also ran our pipeline on phages that target other host genera, thereby expanding our dataset of RBPs and their predicted structures to 19 081 RBPs from 8525 phages across 238 host genera. Supplementary Figure S3a shows the distribution of the RBP lengths; the mean length is 565 amino acids, and the median is 507 amino acids. Supplementary Figure S3b shows the distribution of ColabFold’s confidence scores in its predicted protein structures. The mean confidence score across all the proteins in our dataset is 78.45%, and the median is 81.69%.

### 2.4 Structure-aware embedding generation

In order to generate the vector representations (embeddings) of the RBPs, we employed SaProt, a structure-aware protein language model that adopts the architecture of ESM-2 (Lin *et al.* 2023) but with the embedding layer modified in view of its structure-aware alphabet. SaProt’s alphabet attempts to capture both sequence and structure information by taking the Cartesian product of the set of all possible residues and Foldseek’s (van Kempen *et al.* 2024) alphabet. Foldseek’s alphabet consists of 20 letters that describe the tertiary interaction between residues and their nearest neighbors; this alphabet was learned via a vector quantized variational autoencoder (van den Oord *et al.* 2017). Formally, suppose we are given a protein of length *n*. Its sequence representation is $\langle r_{1},r_{2},r_{3},\ldots ,r_{n}\rangle$, where *r_i_* is its *i*th residue. Foldseek encodes its structure information into an *n*-letter string $\langle f_{1},f_{2},f_{3},\ldots ,f_{n}\rangle$. The input to SaProt is $\langle \left(r_{1},f_{1}\right),\left(r_{2},f_{2}\right),\left(r_{3},f_{3}\right),\ldots ,\left(r_{n},f_{n}\right)\rangle$. To obtain a fixed-length embedding, we averaged the last layer’s hidden states over the sequence length, resulting in a 1280-long dense vector representation (structure-aware embedding) for each RBP.

We used the 650-million-parameter version of SaProt that was pretrained on 40 million structures from AlphaFold DB (Varadi *et al.* 2022).

### 2.5 Sequence similarity-based training and test set construction

We investigated model performance at different train-versus-test sequence similarity thresholds. A lower train-versus-test sequence similarity indicates that the sequences in the training set are more dissimilar to those in the test set.

To this end, we used CD-HIT (Fu *et al.* 2012) to group the RBPs into clusters at a similarity threshold *s* and assign a representative sequence per cluster. Let *R* be the set of representative sequences with class labels among the ESKAPEE genera. We split *R* into two sets *D*_1_ and *D*_2_, with 70% of the sequences assigned to *D*_1_ and the remaining 30% to *D*_2_; this splitting was stratified based on the class sizes. Clusters with representative sequences in *D*_1_ were assigned to the training set, while those with representative sequences in *D*_2_ were assigned to the test set. We also randomly sampled RBPs with class labels outside the ESKAPEE genera and added these to our test set; the number of RBPs sampled is equal to the size of the smallest ESKAPEE genus in the test set. The rationale for the inclusion of these RBPs is for the performance evaluation to reflect scenarios where our model is fed with an RBP from a phage infecting a host outside our genera of interest.

Finally, in order to mitigate class imbalance (Supplementary Table S2), we performed data augmentation via SMOTE-Tomek (Batista *et al.* 2003). Our training and test set statistics are reported in Table 2; the per-class breakdown is given in Supplementary Table S2.

**Table 2.** Training and test set statistics.

Sequence similarity *s*	Training set size before SMOTE-TOMEK	Training set size after SMOTE-Tomek	Test set size
100%	5338	16 942	2340
80%	4865	14 822	2822
60%	4523	13 099	3153
40%	4465	11 662	3203

### 2.6 Classifier building

We defined phage–host interaction prediction as a multiclass classification task where the input is the SaProt embedding of a given RBP and the output is one of ESKAPEE host genera.

**Figure 2.**
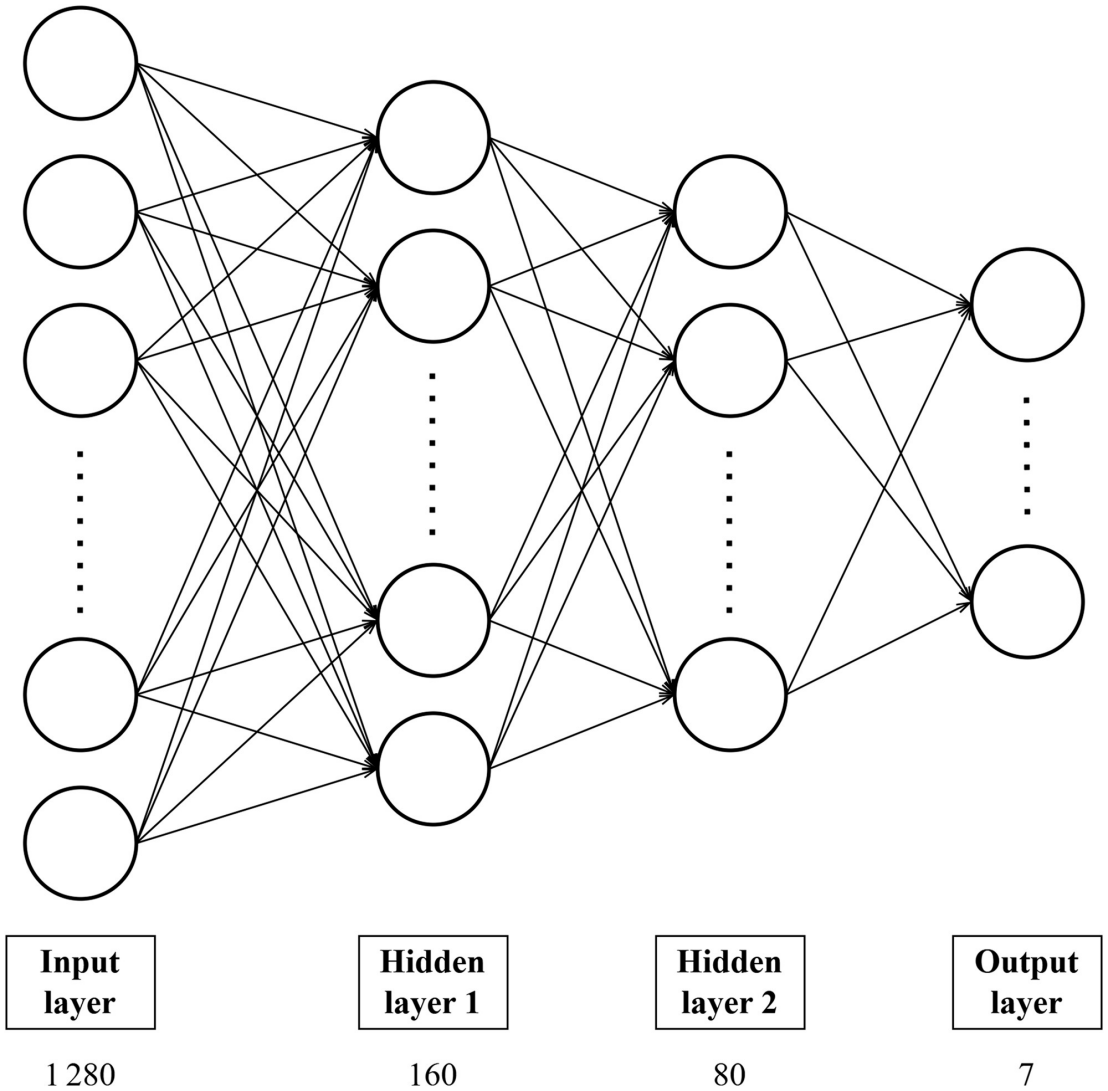
PHIStruct classifier architecture. The number below the label of each layer denotes the size of that layer.

To this end, we built a multilayer perceptron with two hidden layers (Fig. 2). The first and second hidden layers have 160 (one-eighth of the size of the input layer) and 80 neurons, respectively. We implemented *L*_2_ regularization and applied dropout with a rate of 0.2 after the first hidden layer. We employed rectified linear unit as the activation function, softmax as the output function, and cross-entropy as the loss function. We trained the model for 200 epochs with Adam (Kingma and Ba 2015) as the optimizer; other training hyperparameters are listed in Table 3. Hyperparameter tuning was done by performing grid search and optimizing the F1 on a validation set constructed by setting aside 10% of our training set; the hyperparameter search space is given in Supplementary Table S3. We call the resulting model PHIStruct.

**Table 3.** PHIStruct training hyperparameters.

Hyperparameter	Value
*L*_2_ regularization strength	$10^{-4}$
Batch size	128
Learning rate	$10^{-3}$
Exponential decay rate for the first moment vector	0.9
Exponential decay rate for the second moment vector	0.999

### 2.7 Performance evaluation

We evaluated the performance of our model in terms of class-averaged metrics: macro-precision, macro-recall, and macro-F1. To account for our model’s confidence in its prediction, we parameterized these metrics based on a confidence threshold *k*. Let *p*_1_ and *p*_2_ be the class probabilities for the model’s highest-ranked and its second-highest-ranked predictions, respectively. The input is classified under the highest-ranked prediction only if $p_{1}-p_{2}\geq k$. In this regard, higher values of *k* prioritize precision, whereas lower values of *k* prioritize recall. We also parameterized the metrics based on the maximum train-versus-test sequence similarity *s*.

Let *C* be the set of ESKAPEE class labels, and let $TP_{c,k,s},\;TN_{c,k,s},\;FP_{c,k,s}$, and $FN_{c,k,s}$ denote the number of true positive, true negative, false positive, and false negative classifications for class c∈C, respectively, at confidence threshold *k* and maximum train-versus-test sequence similarity *s*. We define the class-averaged (macro) metrics formally in Equations (1)–(3).

(1) $$\mathrm{Macro}–\mathrm{Precisio}n_{k,s}=\frac{1}{\left|C\right|}\sum_{c\in C}\frac{TP_{c,k,s}}{TP_{c,k,s}+FP_{c,k,s}}$$

(2) $$\mathrm{Macro}–\mathrm{Recal}l_{k,s}=\frac{1}{\left|C\right|}\sum_{c\in C}\frac{TP_{c,k,s}}{TP_{c,k,s}+FN_{c,k,s}}$$

(3) $$\begin{array}{cc} & \mathrm{Macro}–F1_{k,s}\\ & =\frac{1}{\left|C\right|}\sum_{c\in C}\frac{2\cdot \frac{TP_{c,k,s}}{TP_{c,k,s}+FP_{c,k,s}}\cdot \frac{TP_{c,k,s}}{TP_{c,k,s}+FN_{c,k,s}}}{\frac{TP_{c,k,s}}{TP_{c,k,s}+FP_{c,k,s}}+\frac{TP_{c,k,s}}{TP_{c,k,s}+FN_{c,k,s}}} \end{array}$$

We additionally evaluated our model’s performance in terms of weighted metrics, with the weights corresponding to the class sizes. Let *N* refer to the total number of samples in the test set, and let $n_{c,s}$ refer to the number of test samples under class c∈C at maximum train-versus-test sequence similarity *s*. We define the weighted metrics formally in Equations (4)–(6).

(4) $$\mathrm{Weighted}–\mathrm{Precisio}n_{k,s}=\frac{1}{N}\sum_{c\in C}n_{c,s}\cdot \frac{TP_{c,k,s}}{TP_{c,k,s}+FP_{c,k,s}}$$

(5) $$\mathrm{Weighted}–\mathrm{Recal}l_{k,s}=\frac{1}{N}\sum_{c\in C}n_{c,s}\cdot \frac{TP_{c,k,s}}{TP_{c,k,s}+FN_{c,k,s}}$$

(6) $$\begin{array}{cc} & \mathrm{Weighted}–F1_{k,s}=\\ & \frac{1}{N}\sum_{c\in C}n_{c,s}\cdot \frac{2\cdot \frac{TP_{c,k,s}}{TP_{c,k,s}+FP_{c,k,s}}\cdot \frac{TP_{c,k,s}}{TP_{c,k,s}+FN_{c,k,s}}}{\frac{TP_{c,k,s}}{TP_{c,k,s}+FP_{c,k,s}}+\frac{TP_{c,k,s}}{TP_{c,k,s}+FN_{c,k,s}}} \end{array}$$

## 3 Results and discussion

### 3.1 At lower train-versus-test sequence similarity, PHIStruct outperforms state-of-the-art machine learning tools that take in receptor-binding proteins as input, as well as sequence alignment-based tools

We benchmarked our tool, PHIStruct, against state-of-the-art machine learning tools that also map receptor-binding proteins to host bacteria: Boeckaerts *et al.*’s (2021) tool, PHIEmbed (Gonzales *et al.* 2023), and Badam and Rao’s (2024) tool. Boeckaerts *et al.*’s (2021) tool is a random forest model that takes in manually feature-engineered genomic and protein sequence properties. Our previous work, PHIEmbed, is a random forest model that takes in ProtT5 (Elnaggar *et al.* 2022) embeddings. Badam and Rao’s (2024) tool is a multilayer perceptron that takes in ESM-1b (Rives *et al.* 2021) embeddings. To ensure a fair comparison, we retrained each of them on our dataset. We also benchmarked PHIStruct against BLASTp, which we ran at an E-value of 0.05 and with BLOSUM62 as the scoring matrix, and against PSI-BLAST, which we ran for up to five iterations at an E-value of 0.05 and a profile inclusion threshold of 0.002; for these sequence alignment-based tools, the class label of the reported top hit was taken as the predicted host genus. We evaluated the tools’ performance across maximum train-versus-test sequence similarity thresholds s=40% to 100% in steps of 20% and across confidence thresholds k=0% to 90% in steps of 10%.

**Figure 3.**
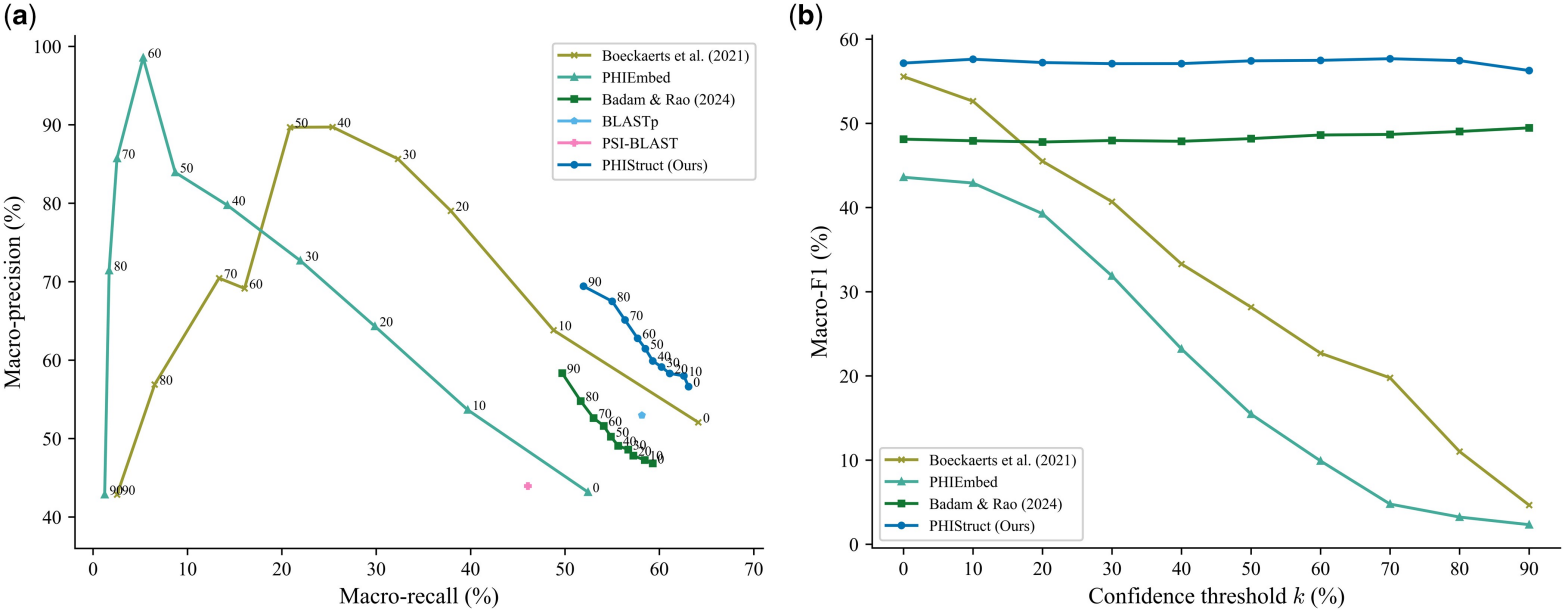
Comparison of the performance of PHIStruct with state-of-the-art machine learning and sequence alignment-based tools that map receptor-binding proteins to host bacteria. The maximum train-versus-test sequence similarity is set to s=40%. Performance is measured in terms of class-averaged (macro) metrics. (a) Precision–recall curves. The label of each point denotes the confidence threshold *k* (%) at which the performance was measured. (b) F1 scores. Higher values of *k* prioritize precision over recall, whereas lower values prioritize recall.

Our experiments showed that PHIStruct presents improvements over these tools, especially at low sequence similarity settings. Its performance gains were most pronounced at s=40% (Fig. 3). It registered a maximum macro-recall of 63.09%, achieving a margin of 17% over PSI-BLAST, 11% over PHIEmbed, 5% over BLASTp, and 4% over Badam and Rao’s (2024) tool and performing competitively with Boeckaerts *et al.*’s (2021) tool. It obtained a maximum macro-precision of 69.43%, outperforming Badam and Rao’s (2024) tool by 11%, BLASTp by 16%, and PSI-BLAST by 25%. Moreover, it recorded the highest maximum macro-F1 at 57.67%, outperforming Boeckaerts *et al.*’s (2021) tool by 2%, BLASTp by 6%, Badam and Rao’s (2024) tool by 8%, PHIEmbed by 14%, and PSI-BLAST by 18%.

Although Boeckaerts *et al.*’s (2021) tool and PHIEmbed had higher maximum macro-precision, this was accompanied by a marked loss in recall. This trade-off was less pronounced with PHIStruct, as reflected in the precision–recall curves (Fig. 3a and Supplementary Figs S4a–S6a) and macro-F1 scores (Fig. 3b and Supplementary Figs S4b–S6b). Across all the tested sequence similarity thresholds, the decrease in its macro-F1 from k=0% to 90% was under 2%. Furthermore, at k>50%, it outperformed the machine learning tool with the next-highest macro-F1, i.e. Badam and Rao’s (2024) tool, by 7%–9%, BLASTp by 5%–6%, and PSI-BLAST by 17%–18%. These results highlight our tool’s ability to make high-confidence predictions without heavily compromising between precision and recall.

PHIStruct also performed competitively at higher sequence similarity thresholds. At s=60%, it recorded a maximum macro-recall of 65.83%, macro-precision of 71.77%, and macro-F1 of 62.95%, outperforming the other machine learning tools in terms of maximum macro-recall and F1 and the sequence alignment-based tools in terms of maximum macro-precision (Supplementary Fig. S4). At s=80%, it obtained a maximum macro-recall of 67.08%, macro-precision of 69.10%, and macro-F1 of 63.73%, placing its maximum macro-recall within 1.6% of the top score (Supplementary Fig. S5). At s=100%, it reported a maximum macro-recall of 81.69%, macro-precision of 87.40%, and macro-F1 of 81.12%, placing its maximum macro-recall within 0.4% of the top-performing machine learning tool’s score and its maximum macro-F1 within 0.1% (Supplementary Fig. S6).

In addition, we evaluated our model’s performance when the metrics were weighted based on the class sizes and found that the results were generally similar to those computed with macro-averaged metrics. At s=40%, PHIStruct achieved the highest maximum weighted recall at 64.23%, outperforming PSI-BLAST by 22%, BLASTp by 9%, PHIEmbed by 18%, and Badam and Rao’s (2024) tool by 15%. It registered a maximum weighted precision of 74.50%, outperforming Badam and Rao’s (2024) tool by 4%, BLASTp by 7%, and PSI-BLAST by 17%. It also obtained a maximum weighted F1 of 63.41%, recording a margin of 4% over BLASTp, 12% over Badam and Rao’s (2024) tool, 14% over PHIEmbed, and 17% over PSI-BLAST. It performed competitively with Boeckaerts *et al.*’s (2021) tool, while also maintaining a more stable F1 (Supplementary Fig. S7).

Moving to the higher sequence similarity thresholds s=60% and 80%, PHIStruct registered the highest maximum weighted recall and F1 among the machine learning tools (Supplementary Figs S8 and S9). At s=100%, its maximum weighted recall and F1 were within 0.3% and 0.1%, respectively, of the top-performing machine learning tool’s scores (Supplementary Fig. S10).

These results demonstrate the utility of PHIStruct for improved prediction of phage–host pairs, especially in use cases where phages of interest have receptor-binding proteins with low sequence similarity to those of known phages. The per-genus results are reported in Supplementary Tables S4–S6.

### 3.2 Visualizing SaProt embeddings reveals local clusters corresponding to the ESKAPEE host genera

**Figure 4.**
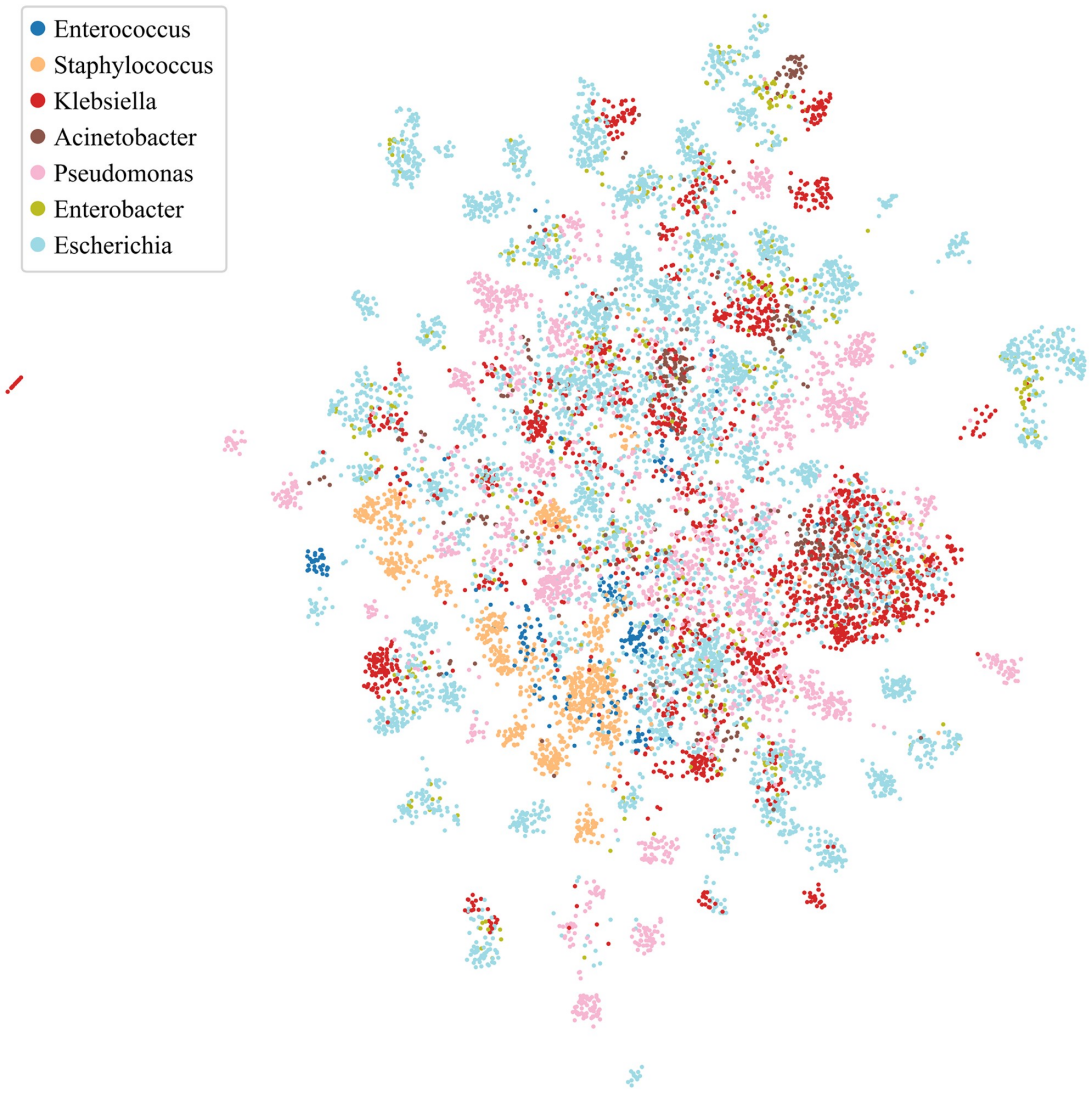
Visualization of the SaProt embeddings using uniform manifold approximation and projection (UMAP). We projected the top 25% SaProt embedding components with the highest importance based on Shapley additive explanations.

We attempted to visualize the SaProt embeddings using uniform manifold approximation and projection or UMAP (McInnes *et al.* 2018). To this end, we ranked the components of the embeddings by their importance based on Shapley additive explanations (Lundberg and Lee 2017) and constructed a vector consisting of the top 25% components with the highest importance, which we then projected onto a 2D space via UMAP. The rationale is to take into account both the input embeddings and the weights assigned by the downstream classifier. As seen in Fig. 4, the formation of local clusters corresponding to the ESKAPEE host genera can be observed. Supplementary Figure S11 shows the UMAP projections of the SaProt embeddings vis-á-vis those of other protein embeddings.

### 3.3 Sequence information and structure information are complementary in improving performance, especially at lower train-versus-test sequence similarity

As discussed in Section 2.4: “Structure-aware embedding generation,” prior to the generation of the structure-aware embedding, SaProt requires a protein to be input as the sequence $\langle \left(r_{1},f_{1}\right),\left(r_{2},f_{2}\right),\ldots ,\left(r_{n},f_{n}\right)\rangle$, where *r_i_* is the *i*th residue and *f_i_* is the corresponding structure (Foldseek) token.

To investigate the impact of incorporating structure information in this input step, we benchmarked different masking strategies: (i) masking all residue tokens, i.e. replacing all $\left(r_{i},f_{i}\right)$ with $\left(\#,f_{i}\right)$, where # is a special token denoting the mask, (ii) masking all structure tokens, i.e. replacing all $\left(r_{i},f_{i}\right)$ with $\left(r_{i},\#\right)$, (iii) masking the structure tokens of low-confidence regions, and (iv) not masking any token (which we employed in PHIStruct). For the third masking strategy, low-confidence regions are defined as those having a pLDDT score below 70%, following the threshold in previous studies (Varadi *et al.* 2022, Su *et al.* 2024).

**Figure 5.**
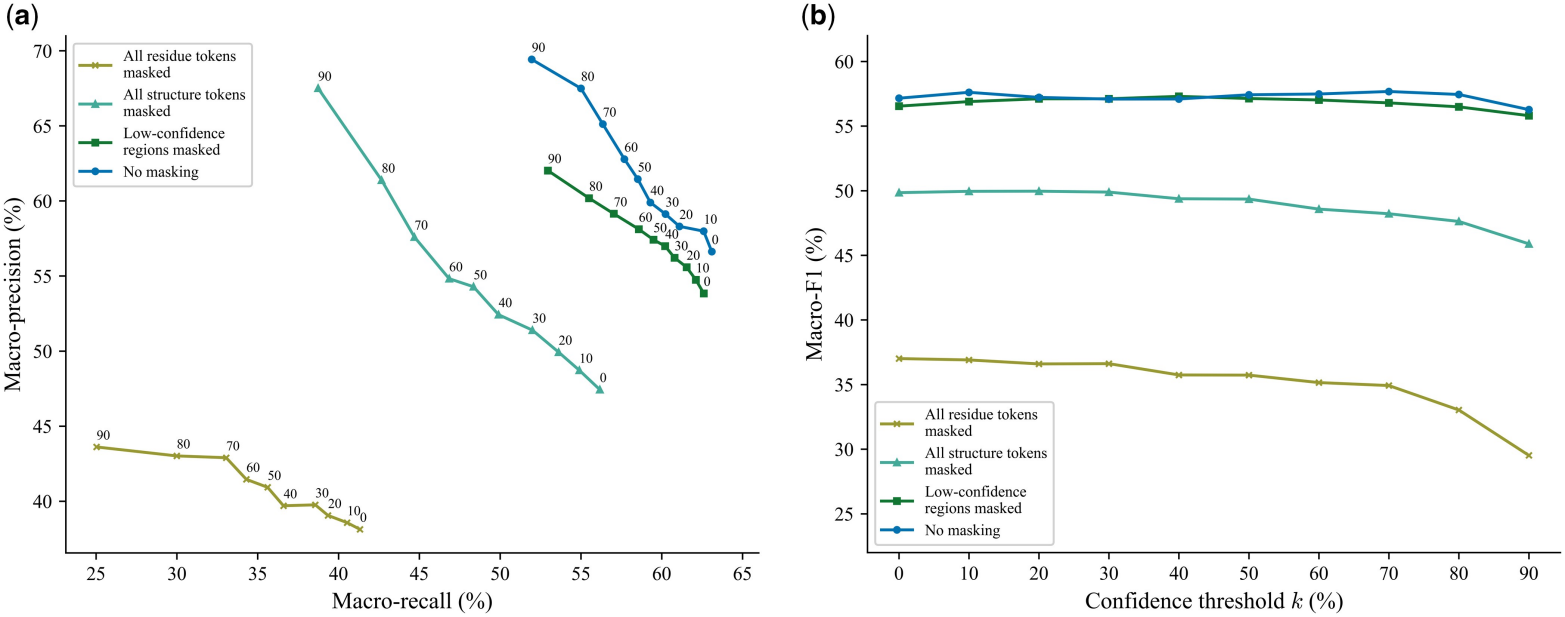
Comparison of the performance of different masking strategies for inputting proteins to SaProt. The maximum train-versus-test sequence similarity is set to s=40%. Performance is measured in terms of class-averaged (macro) metrics. (a) Precision–recall curves. The label of each point denotes the confidence threshold *k* (%) at which the performance was measured. (b) F1 scores. Higher values of *k* prioritize precision over recall, whereas lower values prioritize recall.

Our experiments showed that including both sequence and structure tokens improves performance, especially at lower train-versus-test sequence similarity thresholds. At s=40%, the highest performance across all three metrics was obtained by not masking any token (Fig. 5). Masking all the structure tokens resulted in a 7%, 2%, and 8% decrease in maximum macro-recall, precision, and F1, respectively, whereas masking all the residue tokens resulted in a 22%, 26%, and 21% decrease in these aforementioned metrics. Not masking any token also returned the highest maximum macro-recall, precision, and F1 at s=60% (Supplementary Fig. S12) and the highest maximum macro-recall and F1 at s=80% (Supplementary Fig. S13). At s=100%, the highest performance across all three metrics was achieved by masking the structure tokens of low-confidence regions (Supplementary Fig. S14).

Moreover, we evaluated the performance when the metrics were weighted based on the class sizes and found that the results were generally similar to those computed with macro-averaged metrics. At s=40%, not masking any token resulted in the highest performance across all three metrics. Masking the structure tokens of low-confidence regions led to a 7% decrease in maximum weighted recall and a 5% decrease in both maximum weighted precision and F1. Masking all the structure tokens led to a 4% decrease in both maximum weighted recall and F1, with the maximum weighted precision staying relatively the same. Masking all the sequence tokens led to a 19%, 17%, and 16% decrease in maximum weighted recall, precision, and F1, respectively (Supplementary Fig. S15). At s=60% and 80%, not masking any token returned the highest maximum weighted recall and F1 (Supplementary Figs S16 and S17). At s=100%, it recorded the highest maximum weighted precision, while the highest maximum weighted recall and F1 were achieved by masking the structure tokens of low-confidence regions; however, the difference in the performance between these two approaches was below 1% for all three metrics (Supplementary Fig. S18).

These results suggest that, as the sequence similarity between an RBP of interest and known RBPs decreases, the model benefits from complementing sequence information with structure-informed signals.

### 3.4 Differences in the receptor-binding protein embeddings in relation to the host genus may be contributing to PHIStruct’s discriminative power

To investigate the differences in the embeddings in relation to the host genus, we paired the RBPs such that the sequence similarity between the RBPs in each pair is below 40%. We then computed the cosine distance between the SaProt embeddings of the RBPs in each pair. Afterwards, for every genus, we constructed two groups, each with 500 randomly sampled RBP pairs. In the first group, both RBPs in each pair target the same genus of interest. In the second group, one of the RBPs in each pair targets the genus of interest, while the other RBP targets a different genus. Performing a Mann–Whitney U test showed that, for all but two genera, the difference between the distributions of the cosine distance values in these two groups is statistically significant at a *P*-value cutoff of 0.05 (Supplementary Table S8).

It is possible that PHIStruct may be capturing these differences in the structure-aware embeddings in relation to the host genus, thereby contributing to the model’s discriminative power.

### 3.5 PHIStruct’s use of structure-aware embeddings outperforms the use of sequence-only embeddings, especially at lower train-versus-test sequence similarity

To investigate the impact of the protein representation (i.e. structure-aware versus sequence-only embeddings), we benchmarked PHIStruct against multilayer perceptron models that take in embeddings from ProtT5 (Elnaggar *et al.* 2022), ESM-1b (Rives *et al.* 2021), and ESM-2 (Lin *et al.* 2023), state-of-the-art sequence-only protein language models used in existing phage–host interaction prediction tools (Gonzales *et al.* 2023, Badam and Rao 2024, Boeckaerts *et al.* 2024). The specific model versions used in our benchmarking experiments are given in Supplementary Table S9. The downstream multilayer perceptron models share the same architecture as that of PHIStruct: their first and second hidden layers have $\frac{L}{8}$ and $\frac{L}{16}$ neurons, respectively, where *L* is the length of the protein embedding. To ensure a fair comparison, we performed hyperparameter tuning for each model.

**Figure 6.**
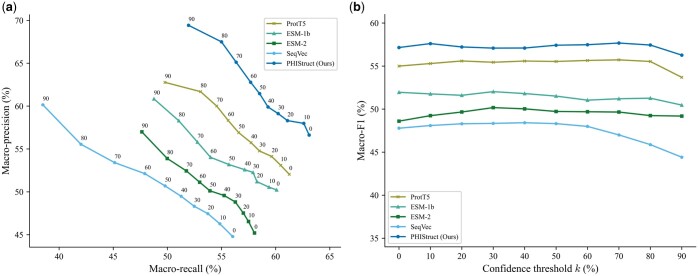
Comparison of the performance of PHIStruct with same-architecture multilayer perceptron models that take in sequence-only embeddings. The maximum train-versus-test sequence similarity is set to s=40%. Performance is measured in terms of class-averaged (macro) metrics. (a) Precision–recall curves. The label of each point denotes the confidence threshold *k* (%) at which the performance was measured. (b) F1 scores. Higher values of *k* prioritize precision over recall, whereas lower values prioritize recall.

Our experiments showed that our use of structure-aware embeddings presents improvements over using sequence-only embeddings, especially at lower train-versus-test sequence similarity thresholds. At s=40%, PHIStruct’s margins over the model with the next-highest performance (i.e. the model using ProtT5) were 7% in terms of maximum macro-precision and 2% in terms of recall and F1 (Fig. 6). At s=60%, it outperformed the model with the next-highest maximum macro-recall (i.e. the model using ProtT5) by 2%, the model with the next-highest maximum macro-precision (i.e. the model using ESM-1b) by 3%, and the model with the next-highest maximum macro-F1 (i.e. the model using ProtT5) by 4% (Supplementary Fig. S19). At s=80%, it obtained the highest maximum macro-recall (Supplementary Fig. S20). At s=100%, it registered the highest maximum macro-precision and F1 (Supplementary Fig. S21).

We also evaluated our model’s performance when the metrics were weighted based on the class sizes and found that the results were generally similar to those computed with macro-averaged metrics. At s=40%, PHIStruct’s margins over the model with the next-highest performance (i.e. the model using ProtT5) were 5%, 4%, and 3% in terms of maximum weighted recall, precision, and F1, respectively (Supplementary Fig. S22). At s=60% and 100%, it registered the highest scores across all three metrics (Supplementary Figs S23 and S25). At s=80%, it registered the highest maximum weighted recall and F1 (Supplementary Fig. S24).

### 3.6 PHIStruct’s use of SaProt embeddings outperforms the use of other structure-aware protein embeddings, especially at lower train-versus-test sequence similarity

To further investigate the impact of the protein representation (i.e. SaProt embeddings versus other structure-aware embeddings), we benchmarked PHIStruct against multilayer perceptron models that share the same architecture as that of PHIStruct but take in embeddings from other structure-aware protein language models: ProstT5 (Heinzinger *et al.* 2024) and PST (Chen *et al.* 2024). ProstT5 adopts the architecture of ProtT5 (Elnaggar *et al.* 2022) and is fine-tuned to convert bidirectionally between protein sequence and structure. PST adopts the architecture of ESM-2 (Lin *et al.* 2023) and augments each self-attention block with a two-layer graph isomorphism network (Xu *et al.* 2019) that serves as a protein structure extractor module. The specific model versions used in our benchmarking experiments are given in Supplementary Table S9. To ensure a fair comparison, we performed hyperparameter tuning for each model.

**Figure 7.**
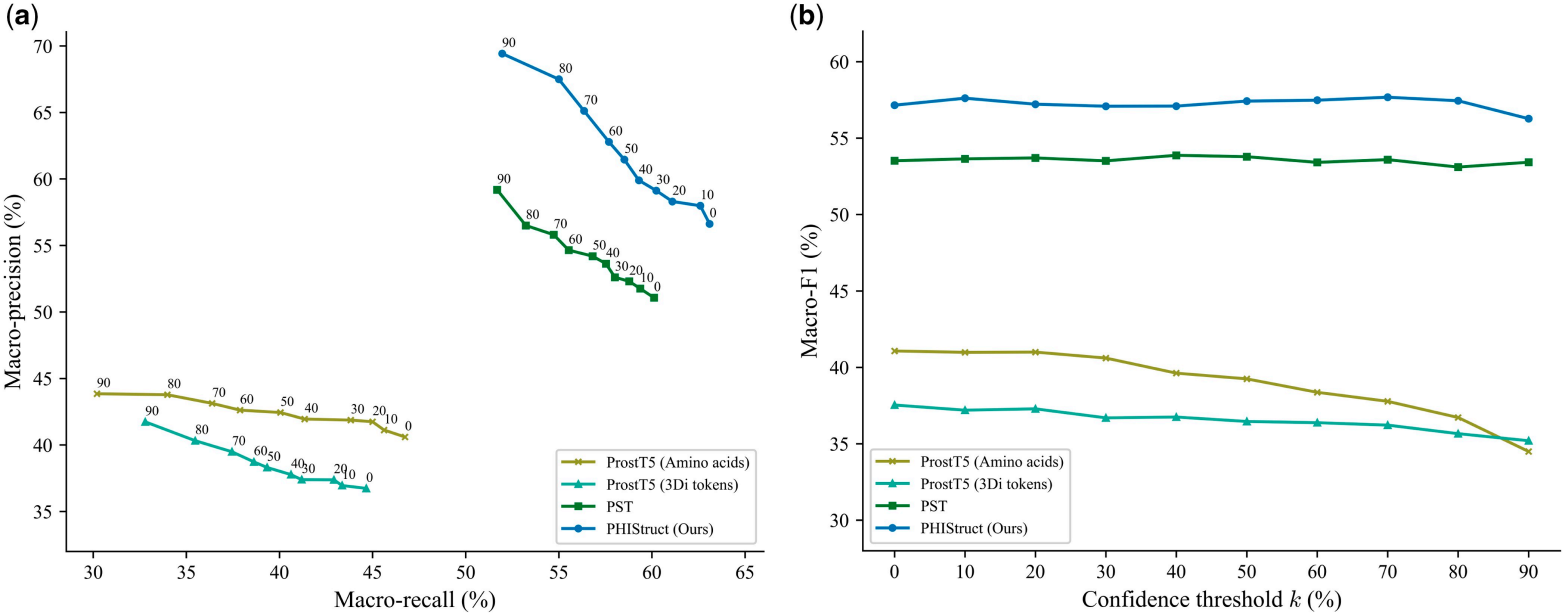
Comparison of the performance of PHIStruct with same-architecture multilayer perceptron models that take in structure-aware protein embeddings other than SaProt. The maximum train-versus-test sequence similarity is set to s=40%. Performance is measured in terms of class-averaged (macro) metrics. (a) Precision–recall curves. The label of each point denotes the confidence threshold *k* (%) at which the performance was measured. (b) F1 scores. Higher values of *k* prioritize precision over recall, whereas lower values prioritize recall.

Our experiments showed that our use of SaProt embeddings presents improvements over using other structure-aware embeddings, especially at lower train-versus-test sequence similarity thresholds. At s=40% and 60%, PHIStruct achieved the highest performance across all three metrics (Fig. 7 and Supplementary Fig. S26). In particular, its performance gains were most pronounced at s=40%, where it outperformed the model with the next-highest performance (i.e. the model using PST) by 3%, 10%, and 4% in terms of maximum macro-recall, precision, and F1, respectively (Fig. 7). At s=80%, it obtained the highest maximum macro-recall and F1, and its maximum macro-precision was within 1.5% of the top score (i.e. from the model using PST) (Supplementary Fig. S27). At s=100%, its maximum macro-recall was within 1.3% of the score of the model using PST and its maximum macro-precision and F1 were both within 0.4% (Supplementary Fig. S28).

Furthermore, we evaluated our model’s performance when the metrics were weighted based on the class sizes and found that the results were generally similar to those computed with macro-averaged metrics. At s=40%, it achieved the highest performance across all three metrics, outperforming the model with the next-highest performance (i.e. the model using PST) by 3% in terms of maximum weighted recall and 5% in terms of maximum weighted precision (Supplementary Fig. S29). At s=60% and 80%, it obtained the highest maximum weighted recall and F1, and its maximum weighted precision was within 0.6% of the score of the model using PST (Supplementary Figs S30 and S31). At s=100%, its performance across all three metrics were within 0.6% of the performance of the model using PST (Supplementary Fig. S32).

### 3.7 PHIStruct’s use of multilayer perceptron outperforms the use of other downstream classifiers, especially at lower train-versus-test sequence similarity

To investigate the impact of the choice of downstream classifier (i.e. multilayer perceptron versus other machine learning models), we benchmarked PHIStruct against a support vector machine and a random forest model that take in the same SaProt embeddings. To ensure a fair comparison, we performed hyperparameter tuning for each model.

**Figure 8.**
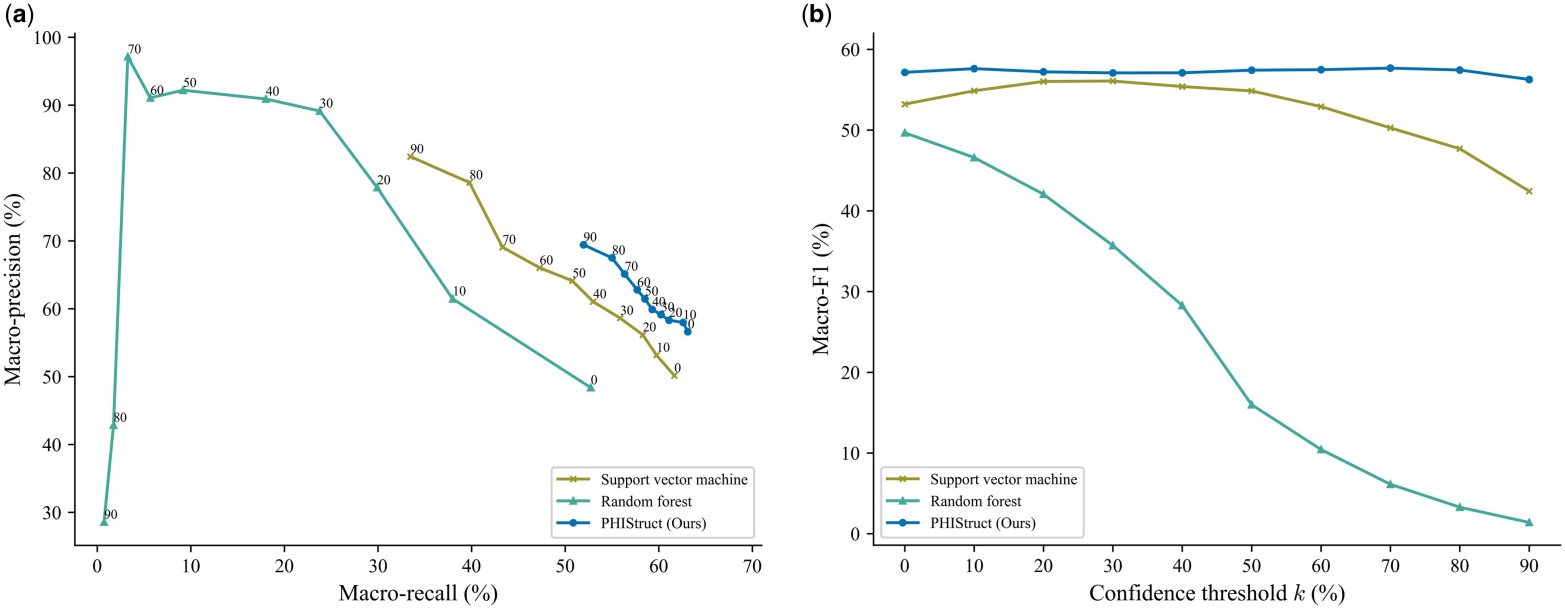
Comparison of the performance of PHIStruct with other downstream classifiers take in the same SaProt embeddings. The maximum train-versus-test sequence similarity is set to s=40%. Performance is measured in terms of class-averaged (macro) metrics. (a) Precision–recall curves. The label of each point denotes the confidence threshold *k* (%) at which the performance was measured. (b) F1 scores. Higher values of *k* prioritize precision over recall, whereas lower values prioritize recall.

Our experiments showed that our use of multilayer perceptron presents improvements over using other machine learning models for the downstream classifier, especially at lower train-versus-test sequence similarity thresholds. From s=40% to 80%, PHIStruct achieved the highest maximum macro-F1 (Fig. 8 and Supplementary Figs S33 and S34). Moreover, at s=40% and s=100%, it also achieved the highest maximum macro-recall (Fig. 8 and Supplementary Fig. S35). At s=60% and s=80%, its maximum macro-recall was within 1.6% of the highest score (Supplementary Figs S33 and S34). Although the random forest model returned the highest maximum macro-precision, this was accompanied by a significant drop in recall, whereas PHIStruct registered the most stable macro-F1 scores across the range of all tested confidence thresholds (Fig. 8 and Supplementary Figs S33–S35).

In addition, we evaluated our model’s performance when the metrics were weighted based on the class sizes and found that the results were generally similar to those computed with macro-averaged metrics. PHIStruct achieved the highest maximum weighted recall and F1, as well as the most stable weighted F1, across all tested sequence similarity thresholds (Supplementary Figs S36–S39).

### 3.8 Room for improvement

**Figure 9.**
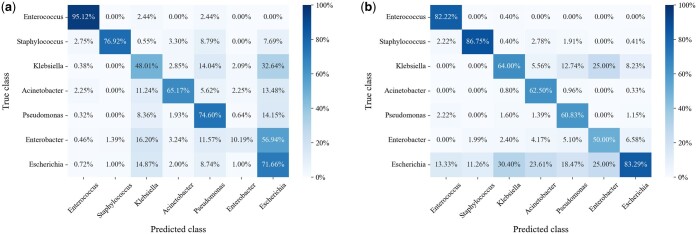
Confusion matrices at maximum train-versus-test sequence similarity s=40%. (a) Confusion matrix at confidence threshold k=0%, normalized over the true class labels. The main diagonal reflects the per-class recall. (b) Confusion matrix at k=90%, normalized over the predicted class labels. The main diagonal reflects the per-class precision. Lower values of *k* prioritize recall over precision, whereas higher values prioritize precision.

We performed error analysis on PHIStruct’s predictions and found that, at lower train-versus-test sequence similarity thresholds, it tends to misclassify phages that infect *Enterobacter* (and *Klebsiella*, albeit to a lesser degree) as infecting *Escherichia* (Fig. 9). This could possibly be related to these three genera belong to the same family, Enterobacteriaceae. The four remaining ESKAPEE genera each belong to different families.

In this regard, possible directions for future work include investigating biological markers that can more strongly distinguish phage–host specificity at lower taxonomic levels. It may also be helpful to develop computational approaches that are explicitly trained to discriminate between biological signals at finer taxonomic resolutions.

Moreover, the identification of receptor-binding proteins is central to our approach. For proteins without existing genome annotations, we employed *in silico* methods to predict whether they are RBPs, and the quality of these predictions is important to our model’s performance. Recent advancements in RBP screening methods (Gonzalez and Scharf 2021, Ge *et al.* 2023) and the possibility of improving computational tools with the inclusion of protein structure information are complementary towards constructing high-quality RBP datasets, from which our model can benefit.

Model interpretability remains a challenge as well. While attention-based methods for probing sequence-only embeddings and amino acid associations have been proposed (Vig *et al.* 2021, Yamaguchi and Saito 2021, Wang *et al.* 2023, Yagimoto *et al.* 2024), extending these techniques to structure-aware tokens and embeddings is an area for further research.

## 4 Conclusion

In this paper, we presented PHIStruct, a deep learning model that capitalizes on structure-aware embeddings of receptor-binding proteins for predicting phage–host interaction. To this end, we predicted the protein structures via ColabFold and generated the structure-aware embeddings via SaProt. Our experiments showed that our proposed approach presents improvements over state-of-the-art methods, especially as the sequence similarity between the training and test set entries decreases. These results highlight the applicability of PHIStruct in improving the prediction of phage–host pairs, especially in settings where newly discovered phages have receptor-binding proteins with low sequence similarity to those of known phages.

With recent investigations showing an association between tailspike proteins and bacterial polysaccharide receptors (Yang *et al.* 2024), future directions include jointly incorporating these proteins, alongside other phage-encoded and host-encoded proteins involved in different stages of the phage infection process.

## Supplementary Material

btaf016_Supplementary_Data

## Data Availability

*The data underlying this article are available* at https://github.com/bioinfodlsu/PHIStruct
