# Supplementary material for: PHIStruct: improving phage–host interaction prediction at low sequence similarity settings using structure-aware protein embeddings
Source: Bioinformatics. 2025 Jan 13;41(1):btaf016. doi: 10.1093/bioinformatics/btaf016 (PMC11783280; doi:10.1093/bioinformatics/btaf016)
Supplement: btaf016_Supplementary_Data [file btaf016_supplementary_data.zip › 8f816_Supplementary Information.pdf]

Supplementary Data

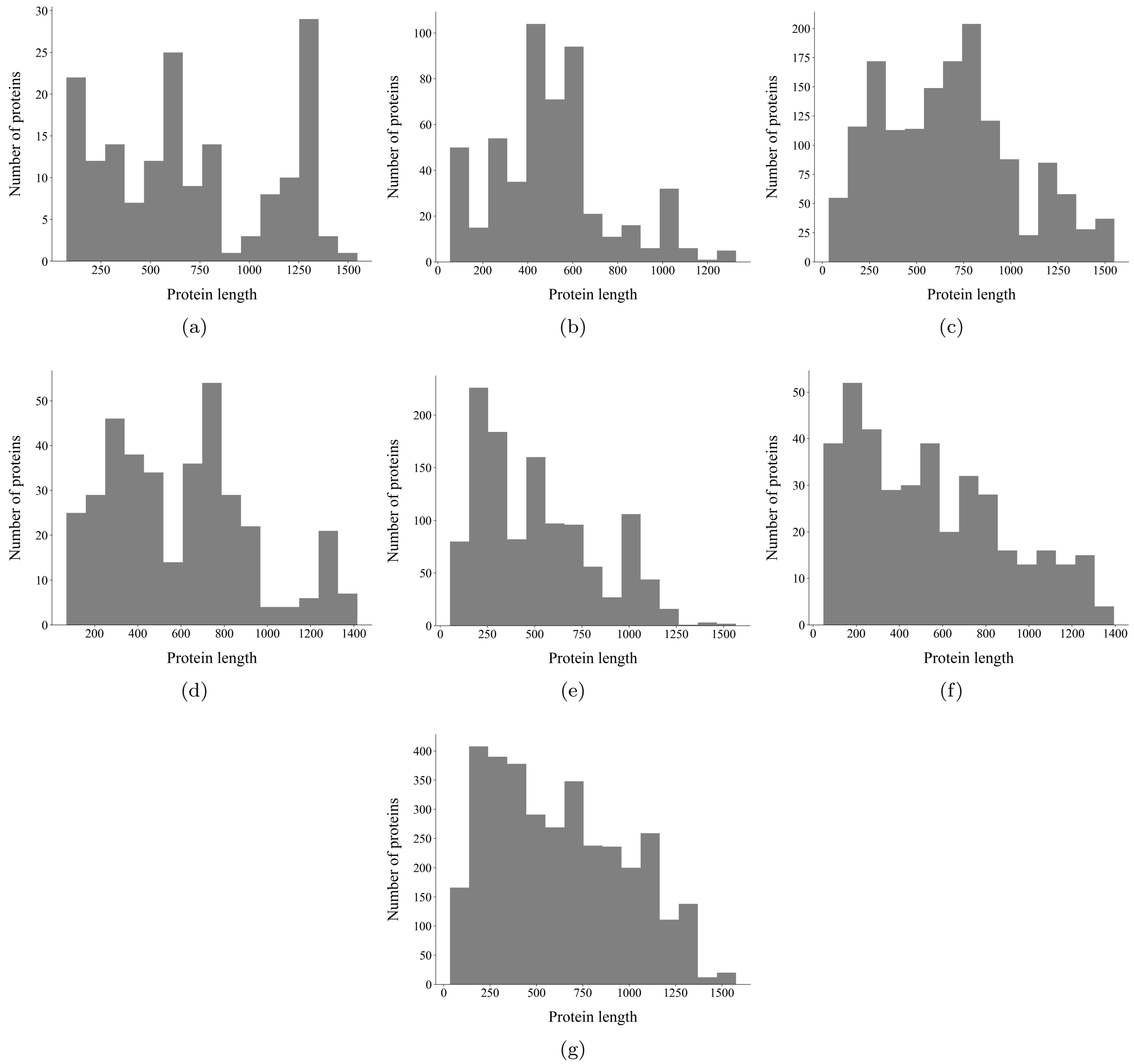

Supplementary Fig. 1: Per-genus distribution of the receptor-binding protein lengths (number of amino acids): (a) *Enterococcus*, (b) *Staphylococcus*, (c) *Klebsiella*, (d) *Acinetobacter*, (e) *Pseudomonas*, (f) *Enterobacter*, and (g) *Escherichia*.

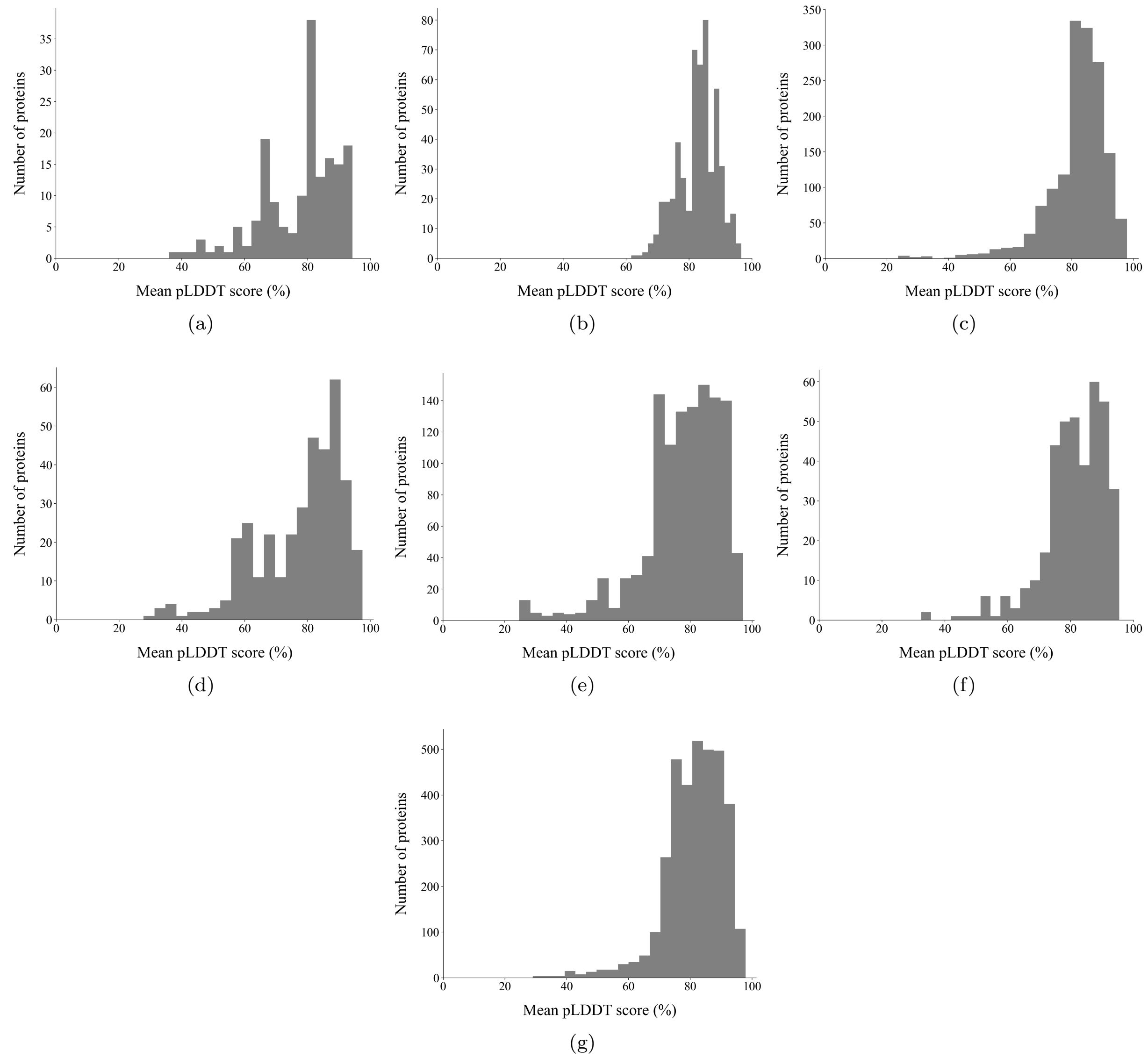

Supplementary Fig. 2: Per-genus distribution of ColabFold's confidence scores in its predicted protein structures: (a) *Enterococcus*, (b) *Staphylococcus*, (c) *Klebsiella*, (d) *Acinetobacter*, (e) *Pseudomonas*, (f) *Enterobacter*, and (g) *Escherichia*. The confidence score for each protein was calculated by taking the mean predicted local distance difference test (pLDDT) scores across its residues.

Supplementary Table 1: ColabFold parameters for protein structure prediction

Parameter	Value
Multiple sequence alignment mode	MMseqs2 (UniRef + Environmental)
Number of models	1
Number of recycles	3
Use of templates	Yes
Amber relaxation parameter	
Max. num. of iterations	200
Max. num. of outer iterations	3
Tolerance	2.39
Stiffness	10.0

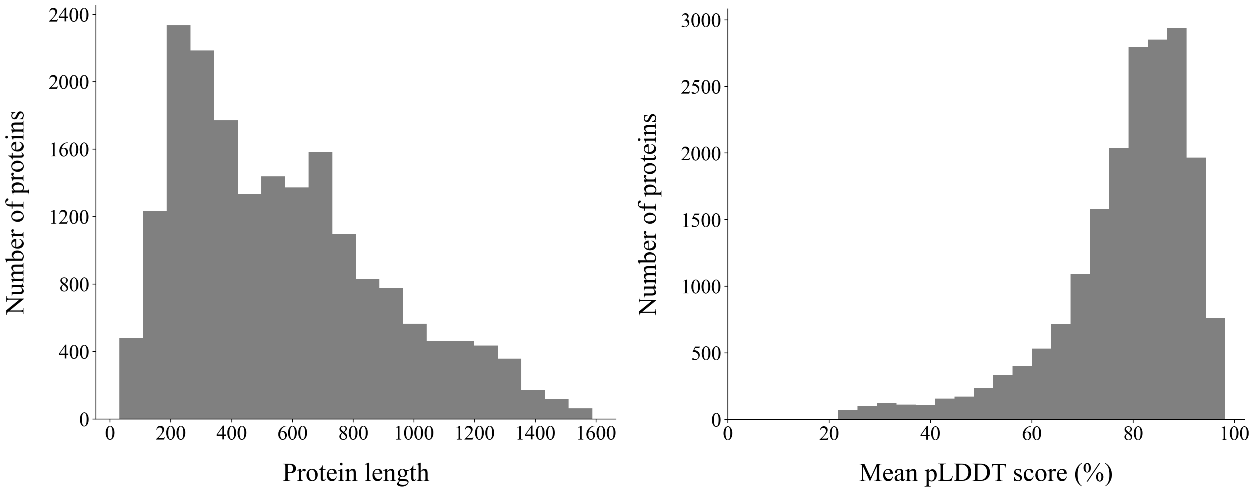

(a) (b)

Supplementary Fig. 3: Dataset characteristics. (a) Distribution of receptor-binding protein lengths (number of amino acids). (b) Distribution of ColabFold's confidence scores in its predicted protein structures. The confidence score for each protein was calculated by taking the mean predicted local distance difference test (pLDDT) scores across its residues.

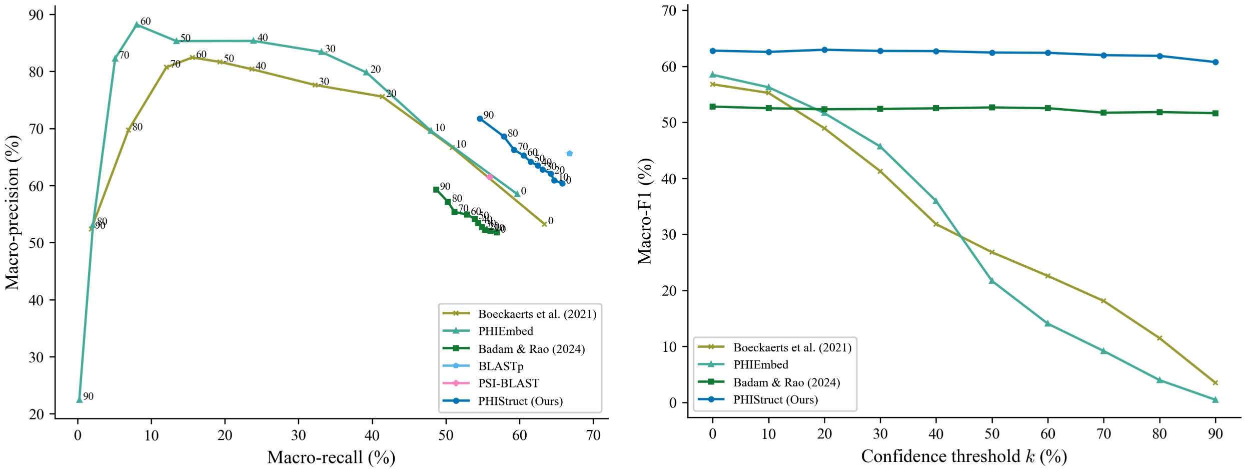

(a) (b)

Supplementary Fig. 4: Comparison of the performance of PHIStruct with state-of-the-art machine learning and sequence alignment-based tools that map receptor-binding proteins to host bacteria. The maximum train-versus-test sequence similarity is set to $s = 60\%$. Performance is measured in terms of class-averaged (macro) metrics. (a) Precision-recall curves. The label of each point denotes the confidence threshold k (%) at which the performance was measured. (b) F1 scores. Higher values of k prioritize precision over recall, whereas lower values prioritize recall.

Supplementary Table 2: Per-class training and test set statistics

Sequence similarity s	Genus	Training set size		Test set size
		Before SMOTE-Tomek	After SMOTE-Tomek	
100%	*Enterococcus*	119	2 424	51
	Staphylococcus	365	2 424	156
	Klebsiella	1 074	2 417	461
	Acinetobacter	258	2 423	111
	Pseudomonas	826	2 422	354
	Enterobacter	272	2 420	116
	Escherichia	2 424	2 412	1 040
	Others	0	0	51
80%	*Enterococcus*	110	2 120	60
	Staphylococcus	349	2 120	172
	Klebsiella	976	2 112	559
	Acinetobacter	260	2 120	109
	Pseudomonas	829	2 119	351
	Enterobacter	221	2 119	167
	Escherichia	2 120	2 112	1 344
	Others	0	0	60
60%	*Enterococcus*	121	1 875	49
	Staphylococcus	364	1 875	157
	Klebsiella	947	1 866	588
	Acinetobacter	248	1 875	121
	Pseudomonas	776	1 874	404
	Enterobacter	192	1 870	196
	Escherichia	1 875	1 864	1 589
	Others	0	0	49
40%	*Enterococcus*	129	1 668	41
	Staphylococcus	339	1 668	182
	Klebsiella	1 008	1 663	527
	Acinetobacter	280	1 668	89
	Pseudomonas	869	1 668	311
	Enterobacter	172	1 666	216
	Escherichia	1 668	1 661	1 796
	Others	0	0	41

Supplementary Table 3: Hyperparameter search space. For the dropout rate, each tested value is given as an ordered pair (x, y), where x and y are the dropout rates after the first and second hidden layers, respectively. The values in bold refer to those adopted by PHIStruct.

Hyperparameters	Search space
Dropout rate	(0, 0), (0.1, 0), **(0.2, 0)**, (0.2, 0.2), (0.3, 0), (0.4, 0), (0.5, 0)
Learning rate	10^{-4}, $\mathbf{10^{-3}}$, 10^{-2}
Batch size	**128**, 256

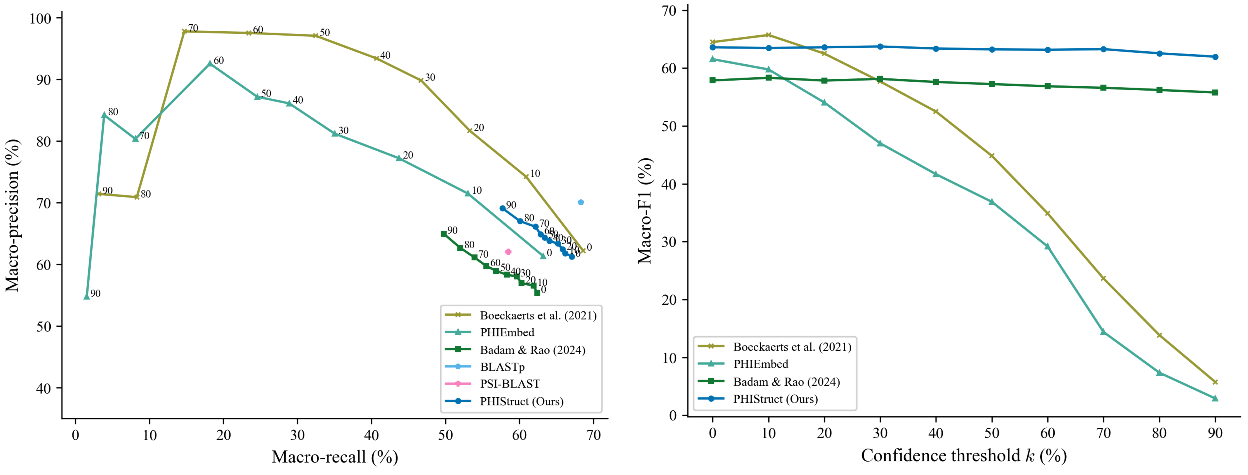

(a) (b)

Supplementary Fig. 5: Comparison of the performance of PHIStruct with state-of-the-art machine learning and sequence alignment-based tools that map receptor-binding proteins to host bacteria. The maximum train-versus-test sequence similarity is set to $s = 80\%$. Performance is measured in terms of class-averaged (macro) metrics. (a) Precision-recall curves. The label of each point denotes the confidence threshold k (%) at which the performance was measured. (b) F1 scores. Higher values of k prioritize precision over recall, whereas lower values prioritize recall.

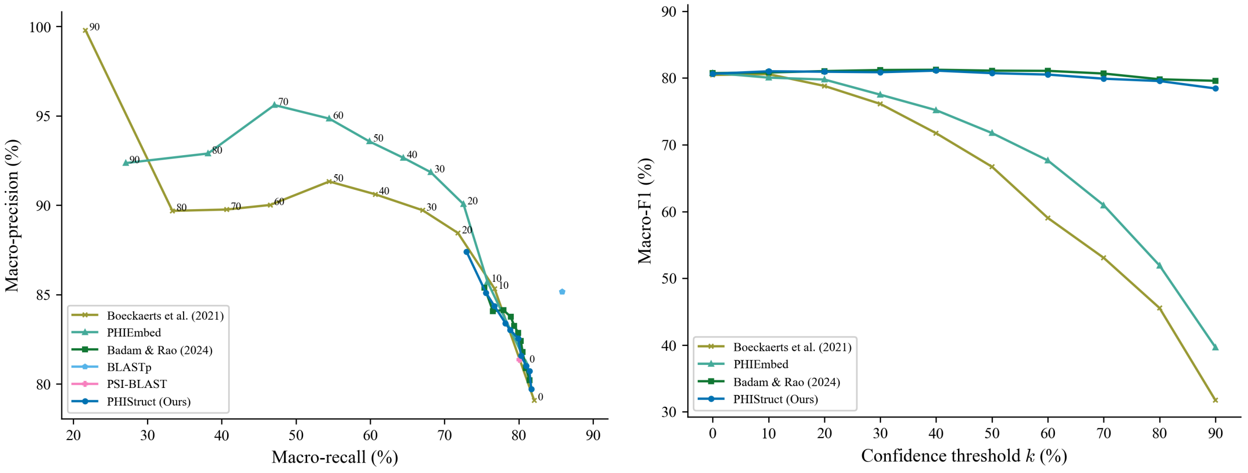

(a) (b)

Supplementary Fig. 6: Comparison of the performance of PHIStruct with state-of-the-art machine learning and sequence alignment-based tools that map receptor-binding proteins to host bacteria. The maximum train-versus-test sequence similarity is set to $s = 100\%$. Performance is measured in terms of class-averaged (macro) metrics. (a) Precision-recall curves. The label of each point denotes the confidence threshold k (%) at which the performance was measured. (b) F1 scores. Higher values of k prioritize precision over recall, whereas lower values prioritize recall.

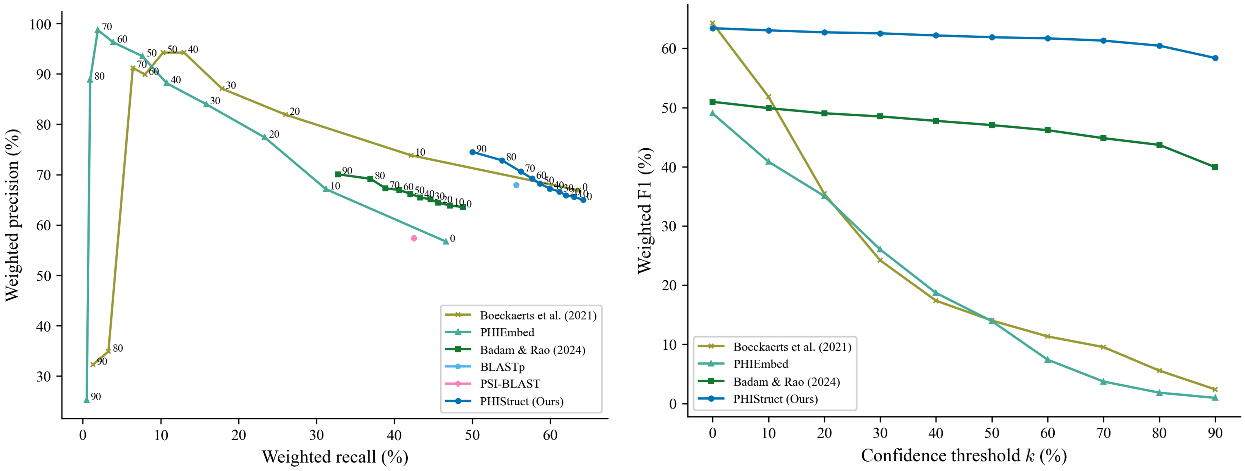

(a) (b)

Supplementary Fig. 7: Comparison of the performance of PHIStruct with state-of-the-art machine learning and sequence alignment-based tools that map receptor-binding proteins to host bacteria. The maximum train-versus-test sequence similarity is set to $s = 40\%$. Performance is measured in terms of weighted metrics. (a) Precision-recall curves. The label of each point denotes the confidence threshold k (%) at which the performance was measured. (b) F1 scores. Higher values of k prioritize precision over recall, whereas lower values prioritize recall.

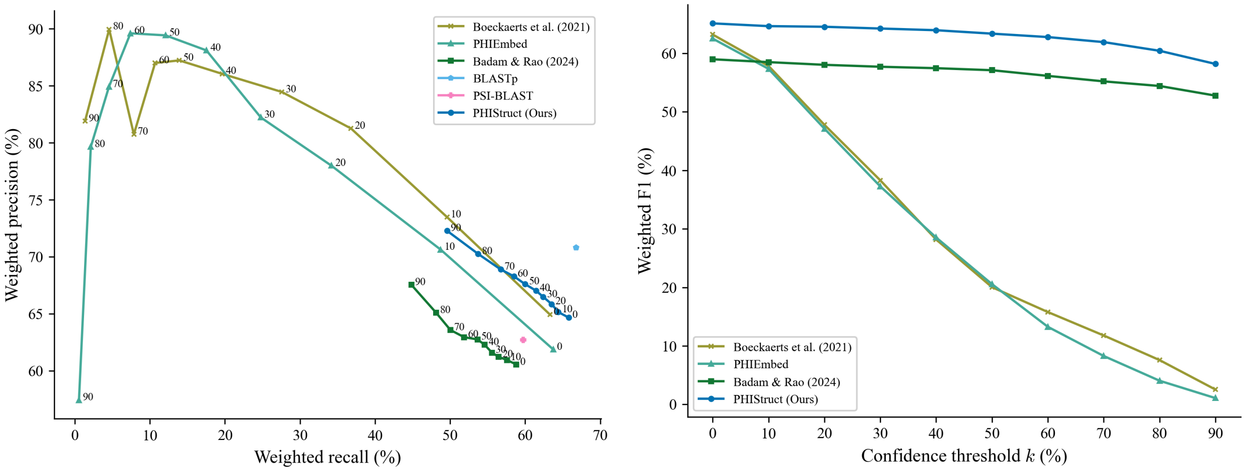

(a) (b)

Supplementary Fig. 8: Comparison of the performance of PHIStruct with state-of-the-art machine learning and sequence alignment-based tools that map receptor-binding proteins to host bacteria. The maximum train-versus-test sequence similarity is set to $s = 60\%$. Performance is measured in terms of weighted metrics. (a) Precision-recall curves. The label of each point denotes the confidence threshold k (%) at which the performance was measured. (b) F1 scores. Higher values of k prioritize precision over recall, whereas lower values prioritize recall.

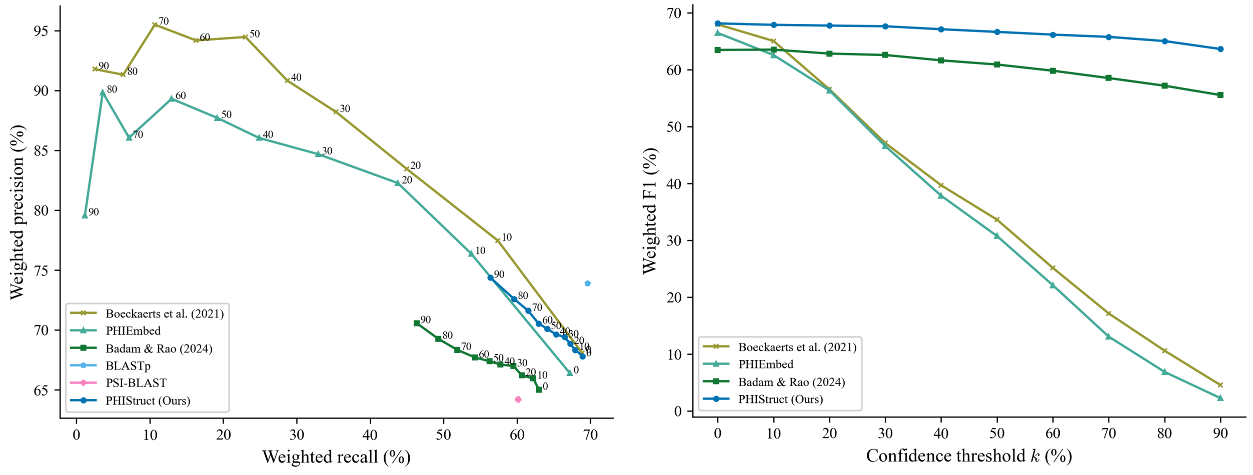

(a) (b)

Supplementary Fig. 9: Comparison of the performance of PHIStruct with state-of-the-art machine learning and sequence alignment-based tools that map receptor-binding proteins to host bacteria. The maximum train-versus-test sequence similarity is set to $s = 80\%$. Performance is measured in terms of weighted metrics. (a) Precision-recall curves. The label of each point denotes the confidence threshold k (%) at which the performance was measured. (b) F1 scores. Higher values of k prioritize precision over recall, whereas lower values prioritize recall.

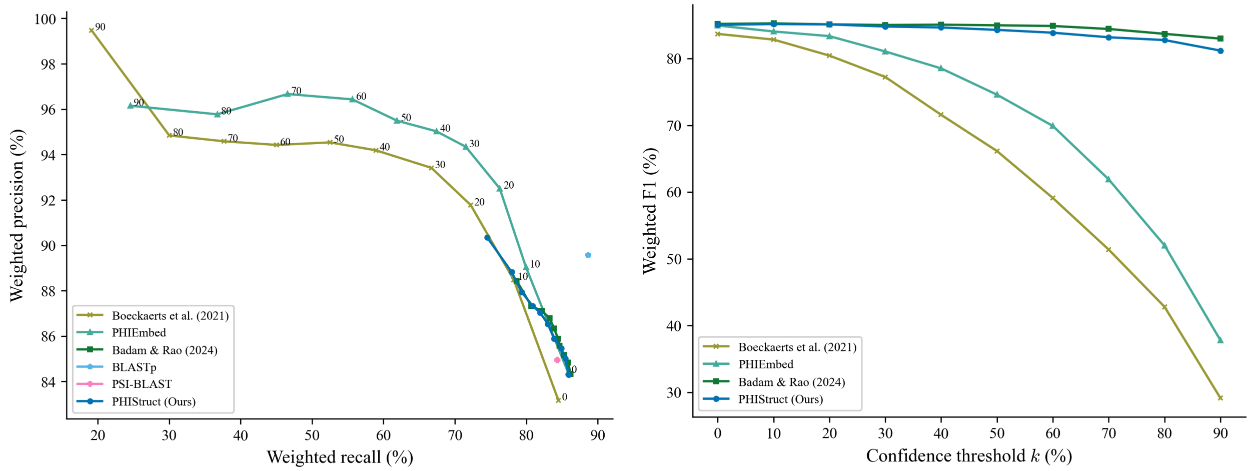

(a) (b)

Supplementary Fig. 10: Comparison of the performance of PHIStruct with state-of-the-art machine learning and sequence alignment-based tools that map receptor-binding proteins to host bacteria. The maximum train-versus-test sequence similarity is set to $s = 100\%$. Performance is measured in terms of weighted metrics. (a) Precision-recall curves. The label of each point denotes the confidence threshold k (%) at which the performance was measured. (b) F1 scores. Higher values of k prioritize precision over recall, whereas lower values prioritize recall.

Supplementary Table 4: Per-class recall of PHIStruct. Lower values of the maximum train-versus-test sequence s indicate that the sequences in the training set are more dissimilar to those in the test set. Lower values of the confidence threshold k (header row) prioritize recall over precision.

		0%	10%	20%	30%	40%	50%	60%	70%	80%	90%
$s = 100\%$	*Enterococcus*	86.27%	86.27%	86.27%	86.27%	86.27%	86.27%	86.27%	84.31%	84.31%	82.35%
	Staphylococcus	97.44%	97.44%	97.44%	96.79%	96.15%	95.51%	95.51%	95.51%	95.51%	95.51%
	Klebsiella	86.98%	86.98%	86.55%	85.68%	85.03%	84.16%	83.51%	82.00%	80.48%	77.22%
	Acinetobacter	81.98%	81.98%	81.98%	81.98%	81.98%	81.98%	81.98%	81.08%	77.48%	74.77%
	Pseudomonas	93.50%	93.50%	93.50%	93.50%	93.50%	93.22%	92.66%	92.09%	92.09%	90.68%
	Enterobacter	38.79%	37.93%	36.21%	34.48%	34.48%	30.17%	28.45%	25.00%	24.14%	19.83%
	Escherichia	86.83%	85.87%	85.00%	83.46%	81.92%	80.58%	79.04%	77.02%	75.10%	70.38%
$s = 80\%$	*Enterococcus*	80.00%	78.33%	78.33%	76.67%	75.00%	75.00%	75.00%	75.00%	71.67%	70.00%
	Staphylococcus	93.60%	93.60%	93.60%	93.60%	93.02%	93.02%	93.02%	92.44%	92.44%	90.12%
	Klebsiella	59.03%	57.25%	56.53%	55.81%	54.92%	54.20%	53.13%	52.06%	50.63%	47.58%
	Acinetobacter	68.81%	68.81%	68.81%	68.81%	68.81%	67.89%	67.89%	67.89%	63.30%	62.39%
	Pseudomonas	69.23%	68.66%	67.81%	66.95%	66.38%	66.10%	65.24%	64.67%	62.96%	60.11%
	Enterobacter	23.95%	22.75%	22.75%	22.16%	19.16%	18.56%	18.56%	17.96%	16.77%	14.97%
	Escherichia	74.93%	73.96%	73.07%	72.17%	70.83%	68.90%	67.11%	64.96%	62.72%	58.78%
$s = 60\%$	*Enterococcus*	83.67%	81.63%	81.63%	79.59%	79.59%	79.59%	79.59%	79.59%	79.59%	75.51%
	Staphylococcus	81.53%	81.53%	81.53%	81.53%	81.53%	81.53%	80.89%	80.89%	80.89%	80.89%
	Klebsiella	45.07%	44.22%	43.88%	43.54%	43.03%	41.50%	39.12%	37.41%	33.84%	29.59%
	Acinetobacter	78.51%	77.69%	77.69%	75.21%	74.38%	73.55%	73.55%	71.90%	71.90%	67.77%
	Pseudomonas	76.49%	75.99%	75.25%	75.00%	74.75%	74.01%	73.02%	71.53%	69.80%	66.34%
	Enterobacter	22.45%	20.92%	19.90%	18.88%	17.35%	15.31%	14.29%	12.24%	11.73%	9.69%
	Escherichia	73.06%	71.05%	69.79%	68.16%	66.77%	65.01%	63.44%	61.36%	57.33%	52.36%
$s = 40\%$	*Enterococcus*	95.12%	95.12%	92.68%	92.68%	92.68%	92.68%	92.68%	92.68%	92.68%	90.24%
	Staphylococcus	76.92%	76.37%	76.37%	76.37%	76.37%	75.82%	74.73%	74.73%	74.73%	71.98%
	Klebsiella	48.01%	47.25%	46.11%	45.54%	44.02%	41.37%	40.23%	37.38%	34.91%	30.36%
	Acinetobacter	65.17%	65.17%	61.80%	58.43%	56.18%	56.18%	55.06%	51.69%	51.69%	50.56%
	Pseudomonas	74.60%	74.60%	73.31%	72.67%	71.70%	70.74%	69.77%	67.85%	65.59%	61.41%
	Enterobacter	10.19%	9.72%	8.33%	7.87%	7.41%	7.41%	6.94%	6.94%	5.09%	2.78%
	Escherichia	71.66%	69.93%	69.04%	68.04%	66.70%	65.42%	64.42%	63.20%	60.30%	56.35%

Supplementary Table 5: Per-class precision of PHIStruct. Lower values of the maximum train-versus-test sequence s indicate that the sequences in the training set are more dissimilar to those in the test set. Higher values of the confidence threshold k (header row) prioritize precision over recall.

		0%	10%	20%	30%	40%	50%	60%	70%	80%	90%
$s = 100\%$	*Enterococcus*	84.62%	84.62%	84.62%	84.62%	84.62%	84.62%	84.62%	84.31%	84.31%	84.00%
	Staphylococcus	93.83%	94.41%	94.41%	94.97%	95.54%	96.13%	96.13%	96.13%	96.75%	97.39%
	Klebsiella	82.00%	82.85%	84.18%	84.76%	86.15%	87.00%	87.90%	88.32%	90.49%	92.47%
	Acinetobacter	87.50%	90.10%	90.10%	91.00%	91.92%	93.81%	94.79%	94.74%	95.56%	97.65%
	Pseudomonas	86.42%	87.57%	87.80%	88.27%	89.46%	90.16%	90.36%	90.56%	91.83%	93.59%
	Enterobacter	35.43%	36.97%	37.17%	38.46%	41.24%	40.23%	40.74%	46.77%	46.67%	56.10%
	Escherichia	88.27%	88.50%	88.84%	89.03%	88.94%	89.24%	89.25%	89.70%	90.08%	90.59%
$s = 80\%$	*Enterococcus*	54.55%	54.65%	54.65%	54.12%	54.88%	54.88%	54.88%	56.25%	55.13%	59.15%
	Staphylococcus	94.15%	94.71%	95.27%	96.99%	96.97%	96.97%	96.97%	97.55%	98.15%	98.10%
	Klebsiella	67.07%	67.51%	69.30%	69.96%	70.41%	71.80%	72.09%	74.42%	76.49%	79.64%
	Acinetobacter	55.97%	56.82%	57.25%	59.52%	61.98%	62.18%	63.25%	64.35%	67.65%	69.39%
	Pseudomonas	56.91%	57.93%	58.48%	59.34%	60.05%	60.42%	60.90%	61.85%	63.14%	65.73%
	Enterobacter	25.64%	26.21%	27.54%	28.91%	27.59%	29.25%	31.00%	32.61%	32.18%	34.25%
	Escherichia	74.37%	74.79%	74.68%	74.79%	74.78%	74.86%	75.23%	75.78%	76.36%	77.45%
$s = 60\%$	*Enterococcus*	71.93%	71.43%	74.07%	73.58%	73.58%	75.00%	76.47%	78.00%	79.59%	84.09%
	Staphylococcus	78.05%	78.53%	79.01%	80.50%	81.53%	82.05%	81.94%	82.47%	83.01%	84.11%
	Klebsiella	50.19%	50.88%	51.70%	52.67%	53.38%	53.63%	53.36%	53.40%	53.64%	55.06%
	Acinetobacter	60.90%	62.25%	63.95%	64.08%	65.22%	66.42%	69.53%	71.31%	75.65%	81.19%
	Pseudomonas	66.45%	67.32%	68.47%	69.82%	70.73%	71.36%	73.02%	73.72%	76.22%	79.76%
	Enterobacter	21.36%	22.04%	23.35%	24.50%	25.37%	25.21%	26.67%	28.24%	34.85%	39.58%
	Escherichia	73.62%	73.89%	74.23%	74.48%	74.77%	75.46%	76.02%	76.59%	77.27%	78.56%
$s = 40\%$	*Enterococcus*	61.90%	65.00%	66.67%	67.86%	70.37%	73.08%	74.51%	79.17%	79.17%	82.22%
	Staphylococcus	86.96%	86.88%	86.88%	86.88%	86.88%	86.79%	86.62%	87.18%	87.18%	86.75%
	Klebsiella	42.31%	43.23%	44.75%	46.24%	47.84%	49.55%	51.21%	53.24%	57.68%	63.24%
	Acinetobacter	43.94%	46.40%	46.61%	46.02%	46.30%	48.08%	50.00%	51.69%	56.10%	62.50%
	Pseudomonas	44.11%	45.05%	45.69%	46.41%	47.45%	49.44%	50.82%	52.75%	55.74%	58.59%
	Enterobacter	40.00%	42.00%	40.00%	42.50%	42.11%	44.44%	46.88%	51.72%	55.00%	50.00%
	Escherichia	77.16%	77.34%	77.55%	77.98%	78.35%	78.81%	79.46%	80.10%	81.61%	82.68%

Supplementary Table 6: Per-class F1 of PHIStruct. Lower values of the maximum train-versus-test sequence s indicate that the sequences in the training set are more dissimilar to those in the test set. Higher values of the confidence threshold k (header row) prioritize precision over recall, whereas lower values prioritize recall.

		0%	10%	20%	30%	40%	50%	60%	70%	80%	90%
	Enterococcus	85.44%	85.44%	85.44%	85.44%	85.44%	85.44%	85.44%	84.31%	84.31%	83.17%
	Staphylococcus	95.60%	95.90%	95.90%	95.87%	95.85%	95.82%	95.82%	95.82%	96.13%	96.44%
	Klebsiella	84.42%	84.87%	85.35%	85.22%	85.59%	85.56%	85.65%	85.04%	85.19%	84.16%
$s = 100\%$	*Acinetobacter*	84.65%	85.85%	85.85%	86.26%	86.67%	87.50%	87.92%	87.38%	85.57%	84.69%
	Pseudomonas	89.82%	90.44%	90.56%	90.81%	91.44%	91.67%	91.49%	91.32%	91.96%	92.11%
	Enterobacter	37.04%	37.45%	36.68%	36.36%	37.56%	34.48%	33.50%	32.58%	31.82%	29.30%
	Escherichia	87.54%	87.16%	86.88%	86.15%	85.29%	84.69%	83.83%	82.88%	81.91%	79.22%
	Enterococcus	64.86%	64.38%	64.38%	63.45%	63.38%	63.38%	63.38%	64.29%	62.32%	64.12%
	Staphylococcus	93.88%	94.15%	94.43%	95.27%	94.96%	94.96%	94.96%	94.93%	95.21%	93.94%
	Klebsiella	62.80%	61.96%	62.27%	62.09%	61.71%	61.77%	61.17%	61.26%	60.93%	59.57%
$s = 80\%$	*Acinetobacter*	61.73%	62.24%	62.50%	63.83%	65.22%	64.91%	65.49%	66.07%	65.40%	65.70%
	Pseudomonas	62.47%	62.84%	62.80%	62.92%	63.06%	63.13%	63.00%	63.23%	63.05%	62.80%
	Enterobacter	24.77%	24.36%	24.92%	25.08%	22.61%	22.71%	23.22%	23.17%	22.05%	20.83%
	Escherichia	74.65%	74.37%	73.86%	73.46%	72.76%	71.76%	70.94%	69.95%	68.87%	66.84%
	Enterococcus	77.36%	76.19%	77.67%	76.47%	76.47%	77.23%	78.00%	78.79%	79.59%	79.57%
	Staphylococcus	79.75%	80.00%	80.25%	81.01%	81.53%	81.79%	81.41%	81.67%	81.94%	82.47%
	Klebsiella	47.49%	47.32%	47.47%	47.67%	47.65%	46.79%	45.14%	44.00%	41.50%	38.50%
$s = 60\%$	*Acinetobacter*	68.59%	69.12%	70.15%	69.20%	69.50%	69.80%	71.49%	71.60%	73.73%	73.87%
	Pseudomonas	71.12%	71.40%	71.70%	72.32%	72.68%	72.66%	73.02%	72.61%	72.87%	72.43%
	Enterobacter	21.89%	21.47%	21.49%	21.33%	20.61%	19.05%	18.60%	17.08%	17.56%	15.57%
	Escherichia	73.34%	72.44%	71.94%	71.18%	70.55%	69.84%	69.16%	68.13%	65.82%	62.84%
	Enterococcus	75.00%	77.23%	77.55%	78.35%	80.00%	81.72%	82.61%	85.39%	85.39%	86.05%
	Staphylococcus	81.63%	81.29%	81.29%	81.29%	81.29%	80.94%	80.24%	80.47%	80.47%	78.68%
	Klebsiella	44.98%	45.15%	45.42%	45.89%	45.85%	45.09%	45.06%	43.92%	43.50%	41.03%
$s = 40\%$	*Acinetobacter*	52.49%	54.21%	53.14%	51.49%	50.76%	51.81%	52.41%	51.69%	53.80%	55.90%
	Pseudomonas	55.44%	56.17%	56.30%	56.64%	57.11%	58.20%	58.81%	59.35%	60.27%	59.97%
	Enterobacter	16.24%	15.79%	13.79%	13.28%	12.60%	12.70%	12.10%	12.24%	9.32%	5.26%
	Escherichia	74.31%	73.45%	73.05%	72.67%	72.06%	71.49%	71.16%	70.65%	69.36%	67.02%

Supplementary Table 7: Results of the Mann-Whitney U test for investigating the structure similarity (in terms of root mean square deviation) of RBPs from phages infecting the same host versus those infecting a different host genus. For every genus, we constructed two groups, each with 500 randomly sampled RBP pairs. In the first group, both RBPs in each pair target the same genus of interest. In the second group, one of the RBPs in each pair targets the genus of interest, while the other RBP targets a different genus.

Genus	p-value
Enterococcus	4.21×10^{-2}
Staphylococcus	5.23×10^{-7}
Klebsiella	9.80×10^{-2}
Acinetobacter	4.99×10^{-3}
Pseudomonas	1.88×10^{-3}
Enterobacter	3.97×10^{-3}
Escherichia	1.59×10^{-2}

Supplementary Table 8: Results of the Mann-Whitney U test for investigating the similarity (in terms of cosine distance) of the embeddings of RBPs from phages infecting the same host versus those infecting a different host genus. For every genus, we constructed two groups, each with 500 randomly sampled RBP pairs. In the first group, both RBPs in each pair target the same genus of interest. In the second group, one of the RBPs in each pair targets the genus of interest, while the other RBP targets a different genus.

Genus	p-value
Enterococcus	2.79×10^{-1}
Staphylococcus	1.41×10^{-36}
Klebsiella	1.49×10^{-4}
Acinetobacter	2.76×10^{-6}
Pseudomonas	8.36×10^{-1}
Enterobacter	5.62×10^{-3}
Escherichia	6.62×10^{-4}

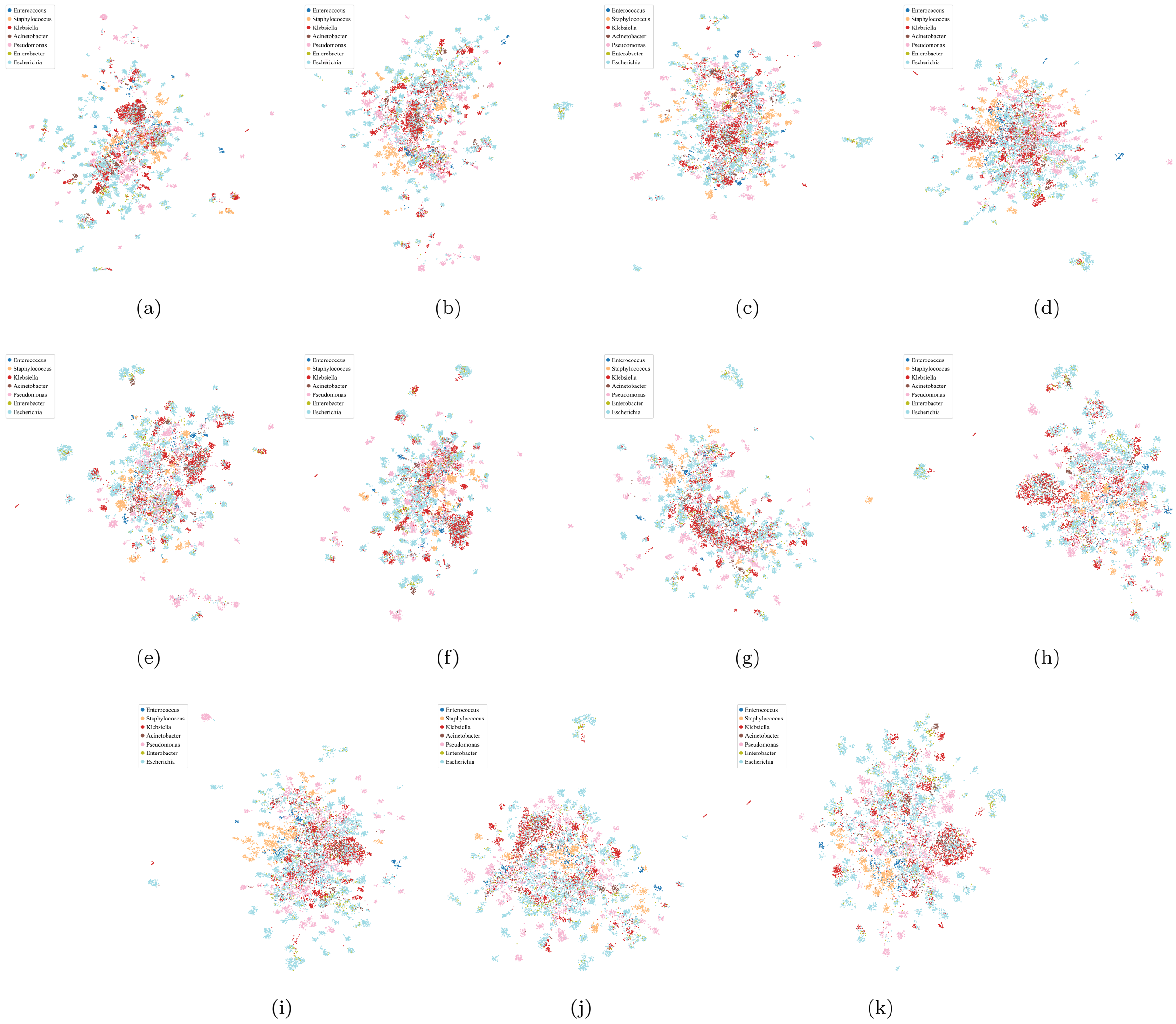

Supplementary Fig. 11: UMAP projections of the top 25% protein embedding components with the highest importance based on Shapley additive explanations. (a) ProtT5. (b) ESM-1b. (c) ESM-2. (d) SeqVec. (e) ProstT5, with amino acids as input. (f) ProstT5, with 3Di tokens as input. (g) PST. (h) SaProt, with all residue tokens masked. (i) SaProt, with all structure tokens masked. (j) SaProt, with low-confidence regions masked. (k) SaProt, with no masking (i.e. the protein representation used by PHIStruct).

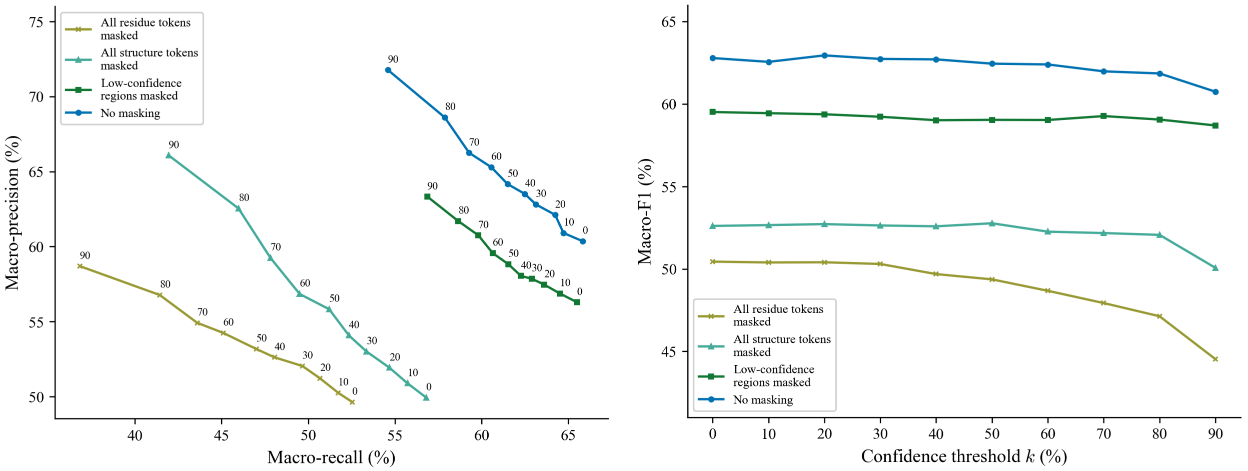

(a) (b)

Supplementary Fig. 12: Comparison of the performance of different masking strategies for inputting proteins to SaProt. The maximum train-versus-test sequence similarity is set to $s = 60\%$. Performance is measured in terms of class-averaged (macro) metrics. (a) Precision-recall curves. The label of each point denotes the confidence threshold k (%) at which the performance was measured. (b) F1 scores. Higher values of k prioritize precision over recall, whereas lower values prioritize recall.

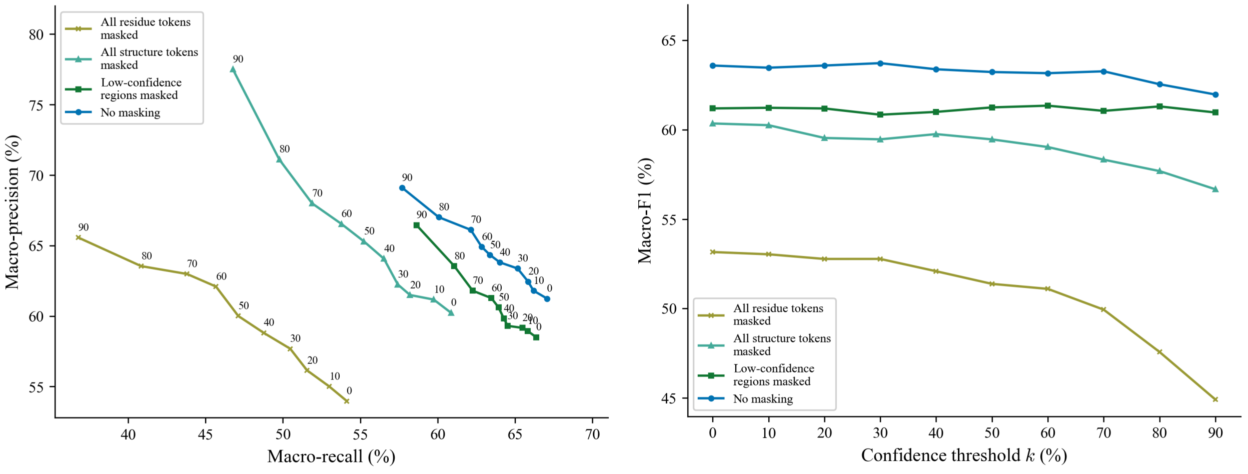

(a) (b)

Supplementary Fig. 13: Comparison of the performance of different masking strategies for inputting proteins to SaProt. The maximum train-versus-test sequence similarity is set to $s = 80\%$. Performance is measured in terms of class-averaged (macro) metrics. (a) Precision-recall curves. The label of each point denotes the confidence threshold k (%) at which the performance was measured. (b) F1 scores. Higher values of k prioritize precision over recall, whereas lower values prioritize recall.

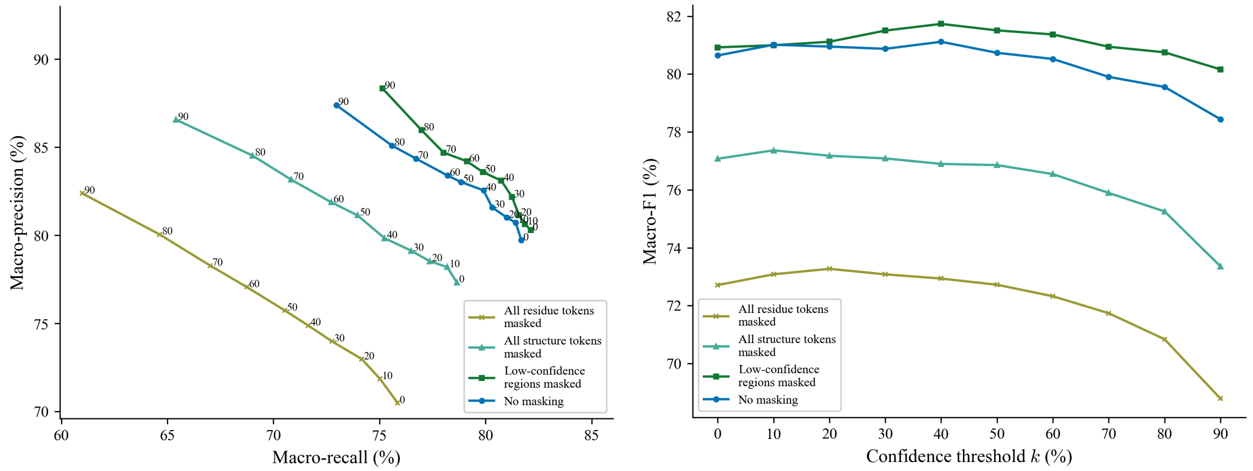

(a) (b)

Supplementary Fig. 14: Comparison of the performance of different masking strategies for inputting proteins to SaProt. The maximum train-versus-test sequence similarity is set to $s = 100\%$. Performance is measured in terms of class-averaged (macro) metrics. (a) Precision-recall curves. The label of each point denotes the confidence threshold k (%) at which the performance was measured. (b) F1 scores. Higher values of k prioritize precision over recall, whereas lower values prioritize recall.

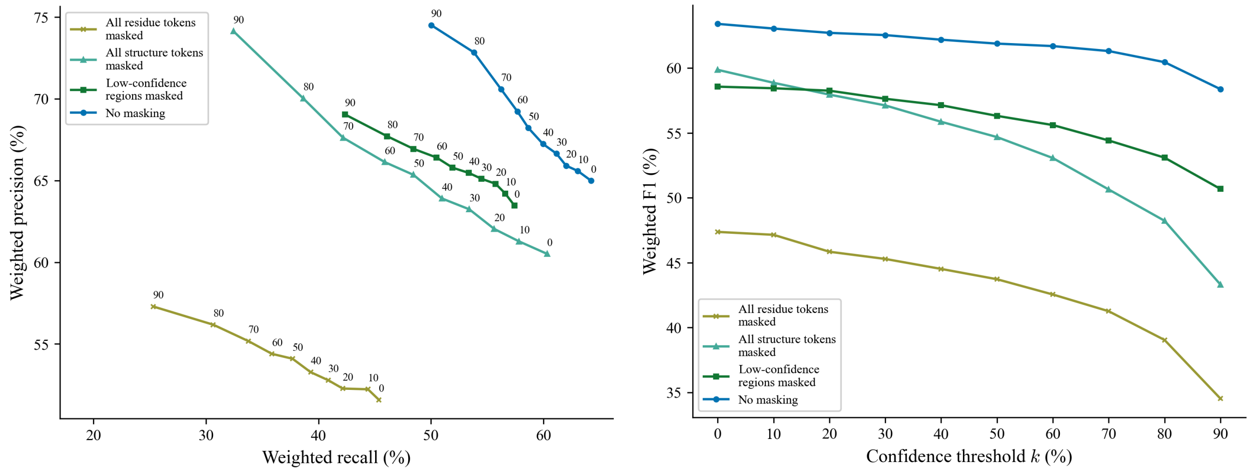

(a) (b)

Supplementary Fig. 15: Comparison of the performance of different masking strategies for inputting proteins to SaProt. The maximum train-versus-test sequence similarity is set to $s = 40\%$. Performance is measured in terms of weighted metrics. (a) Precision-recall curves. The label of each point denotes the confidence threshold k (%) at which the performance was measured. (b) F1 scores. Higher values of k prioritize precision over recall, whereas lower values prioritize recall.

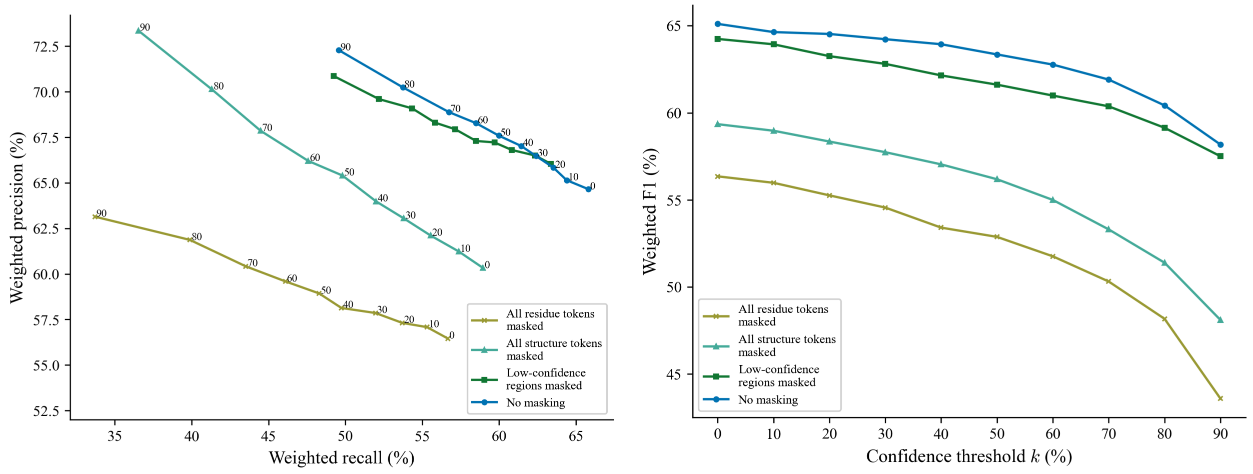

(a) (b)

Supplementary Fig. 16: Comparison of the performance of different masking strategies for inputting proteins to SaProt. The maximum train-versus-test sequence similarity is set to $s = 60\%$. Performance is measured in terms of weighted metrics. (a) Precision-recall curves. The label of each point denotes the confidence threshold k (%) at which the performance was measured. (b) F1 scores. Higher values of k prioritize precision over recall, whereas lower values prioritize recall.

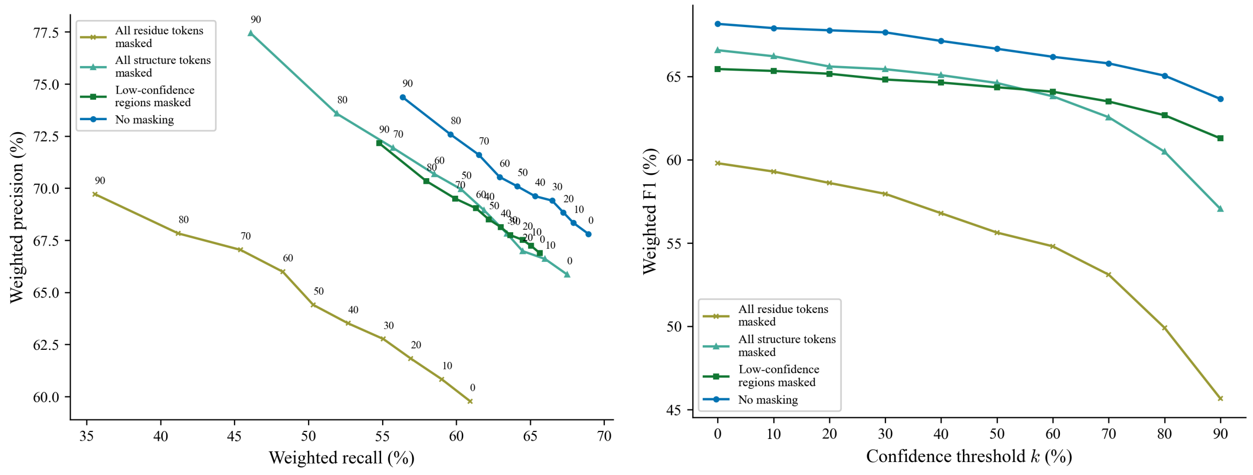

(a) (b)

Supplementary Fig. 17: Comparison of the performance of different masking strategies for inputting proteins to SaProt. The maximum train-versus-test sequence similarity is set to $s = 80\%$. Performance is measured in terms of weighted metrics. (a) Precision-recall curves. The label of each point denotes the confidence threshold k (%) at which the performance was measured. (b) F1 scores. Higher values of k prioritize precision over recall, whereas lower values prioritize recall.

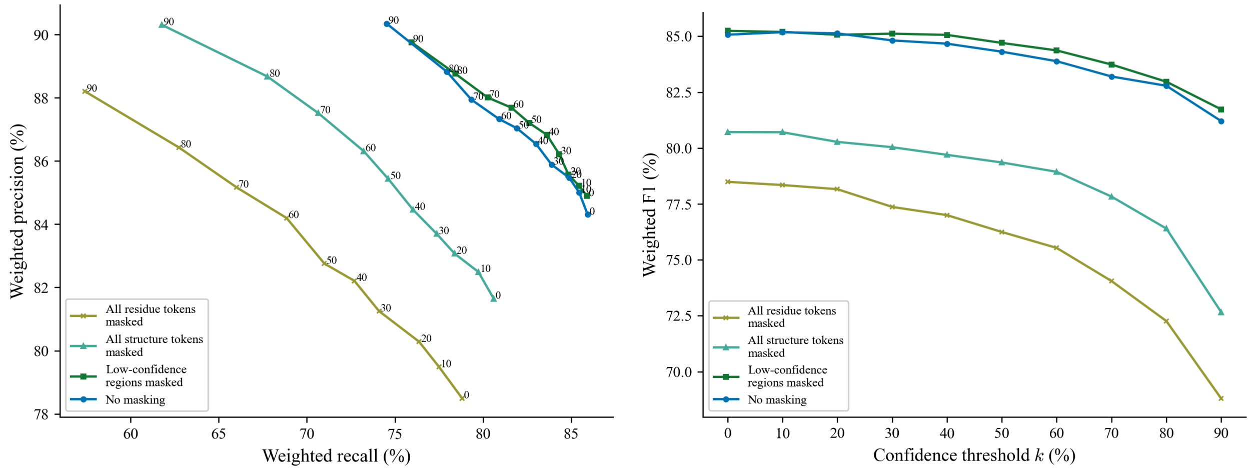

(a) (b)

Supplementary Fig. 18: Comparison of the performance of different masking strategies for inputting proteins to SaProt. The maximum train-versus-test sequence similarity is set to $s = 100\%$. Performance is measured in terms of weighted metrics. (a) Precision-recall curves. The label of each point denotes the confidence threshold k (%) at which the performance was measured. (b) F1 scores. Higher values of k prioritize precision over recall, whereas lower values prioritize recall.

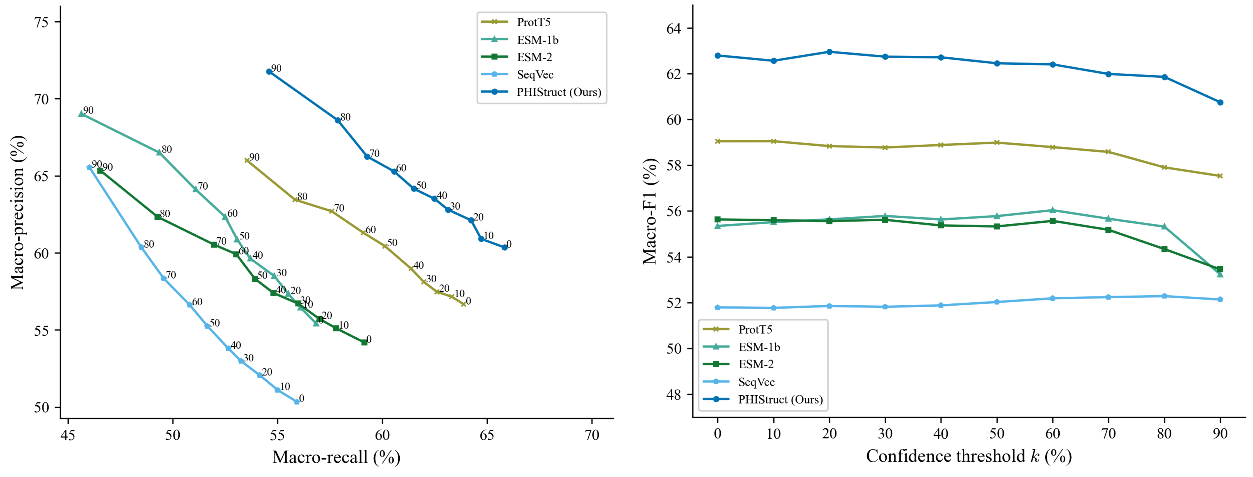

(a) (b)

Supplementary Fig. 19: Comparison of the performance of PHIStruct with same-architecture multilayer perceptron models that take in sequence-only embeddings. The maximum train-versus-test sequence similarity is set to $s = 60\%$. Performance is measured in terms of class-averaged (macro) metrics. (a) Precision-recall curves. The label of each point denotes the confidence threshold k (%) at which the performance was measured. (b) F1 scores. Higher values of k prioritize precision over recall, whereas lower values prioritize recall.

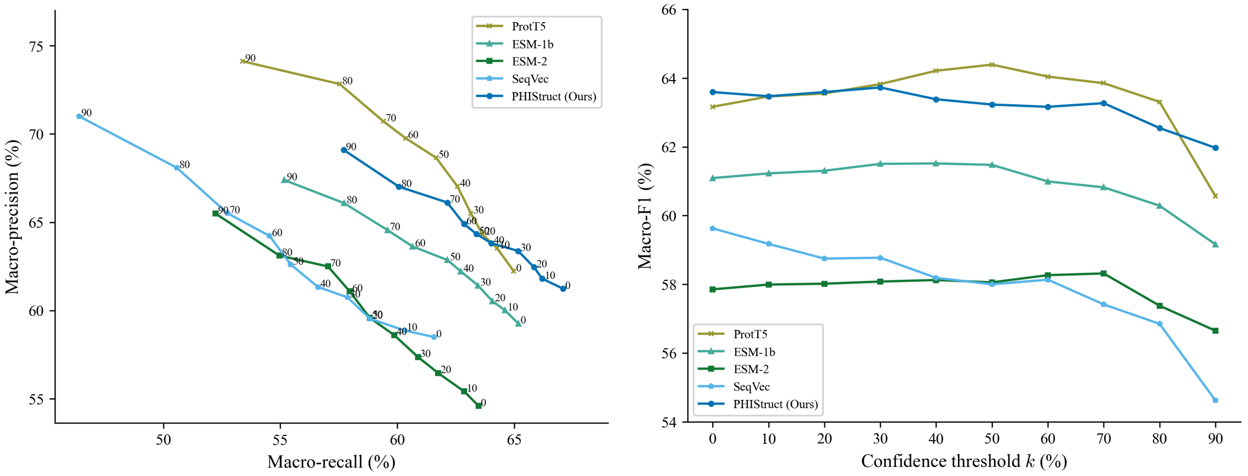

(a) (b)

Supplementary Fig. 20: Comparison of the performance of PHIStruct with same-architecture multilayer perceptron models that take in sequence-only embeddings. The maximum train-versus-test sequence similarity is set to $s = 80\%$. Performance is measured in terms of class-averaged (macro) metrics. (a) Precision-recall curves. The label of each point denotes the confidence threshold k (%) at which the performance was measured. (b) F1 scores. Higher values of k prioritize precision over recall, whereas lower values prioritize recall.

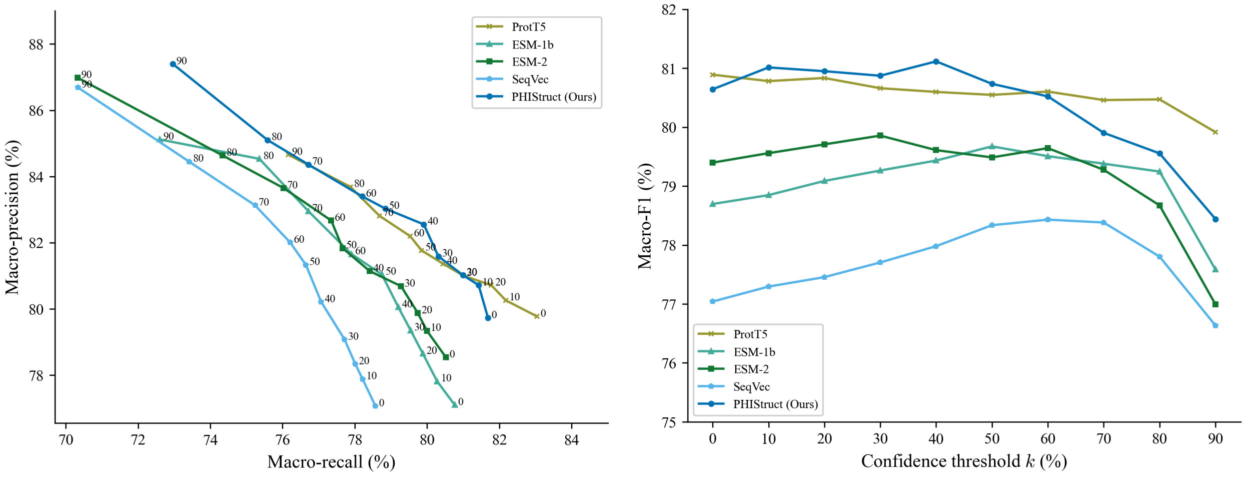

(a) (b)

Supplementary Fig. 21: Comparison of the performance of PHIStruct with same-architecture multilayer perceptron models that take in sequence-only embeddings. The maximum train-versus-test sequence similarity is set to $s = 100\%$. Performance is measured in terms of class-averaged (macro) metrics. (a) Precision-recall curves. The label of each point denotes the confidence threshold k (%) at which the performance was measured. (b) F1 scores. Higher values of k prioritize precision over recall, whereas lower values prioritize recall.

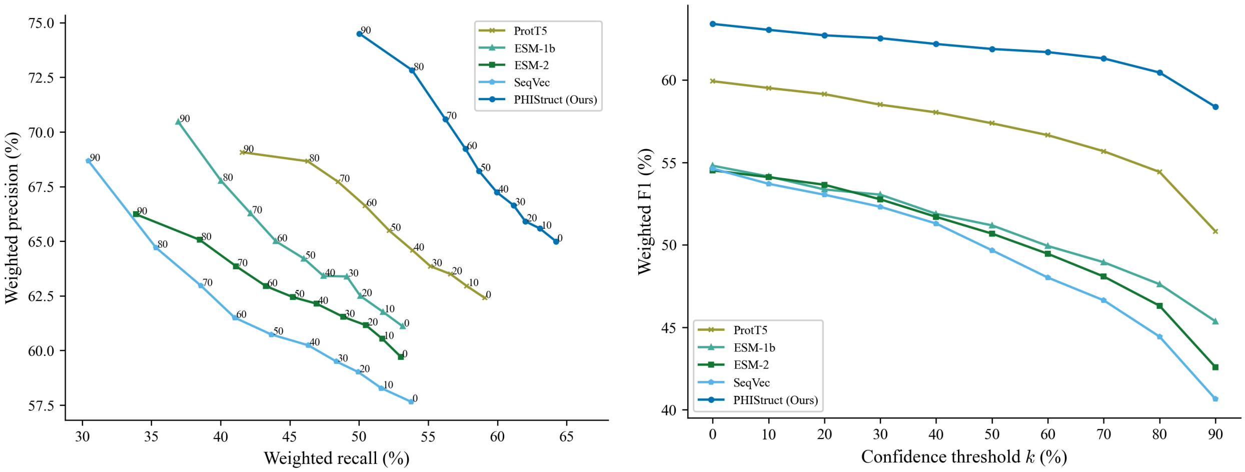

(a) (b)

Supplementary Fig. 22: Comparison of the performance of PHIStruct with same-architecture multilayer perceptron models that take in sequence-only embeddings. The maximum train-versus-test sequence similarity is set to $s = 40\%$. Performance is measured in terms of weighted metrics. (a) Precision-recall curves. The label of each point denotes the confidence threshold k (%) at which the performance was measured. (b) F1 scores. Higher values of k prioritize precision over recall, whereas lower values prioritize recall.

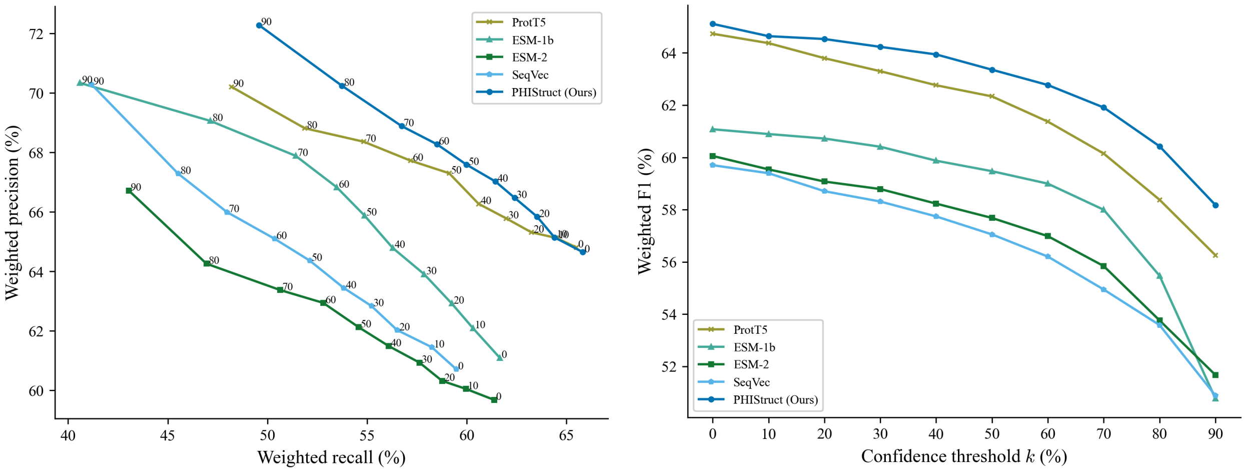

(a) (b)

Supplementary Fig. 23: Comparison of the performance of PHIStruct with same-architecture multilayer perceptron models that take in sequence-only embeddings. The maximum train-versus-test sequence similarity is set to $s = 60\%$. Performance is measured in terms of weighted metrics. (a) Precision-recall curves. The label of each point denotes the confidence threshold k (%) at which the performance was measured. (b) F1 scores. Higher values of k prioritize precision over recall, whereas lower values prioritize recall.

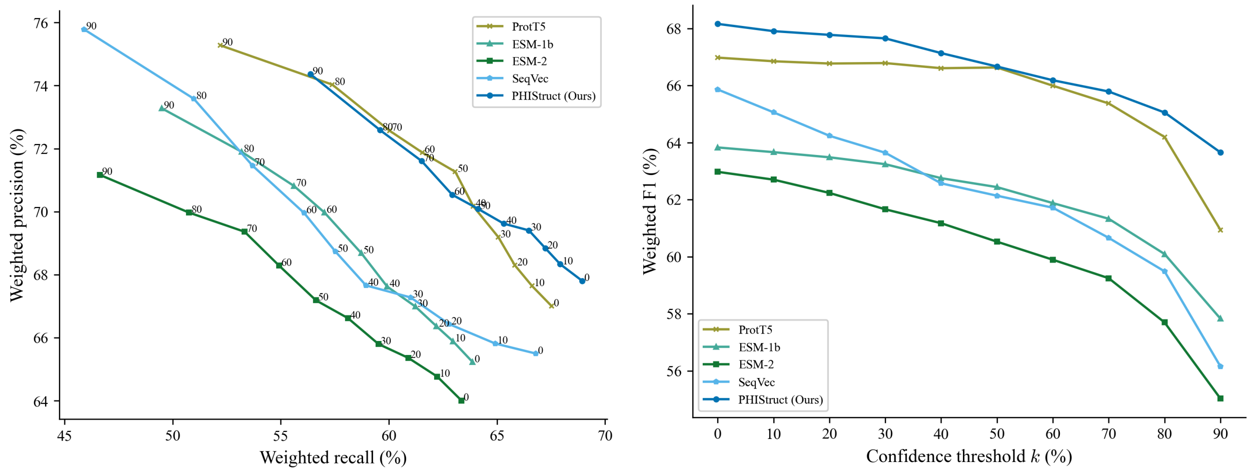

(a) (b)

Supplementary Fig. 24: Comparison of the performance of PHIStruct with same-architecture multilayer perceptron models that take in sequence-only embeddings. The maximum train-versus-test sequence similarity is set to $s = 80\%$. Performance is measured in terms of weighted metrics. (a) Precision-recall curves. The label of each point denotes the confidence threshold k (%) at which the performance was measured. (b) F1 scores. Higher values of k prioritize precision over recall, whereas lower values prioritize recall.

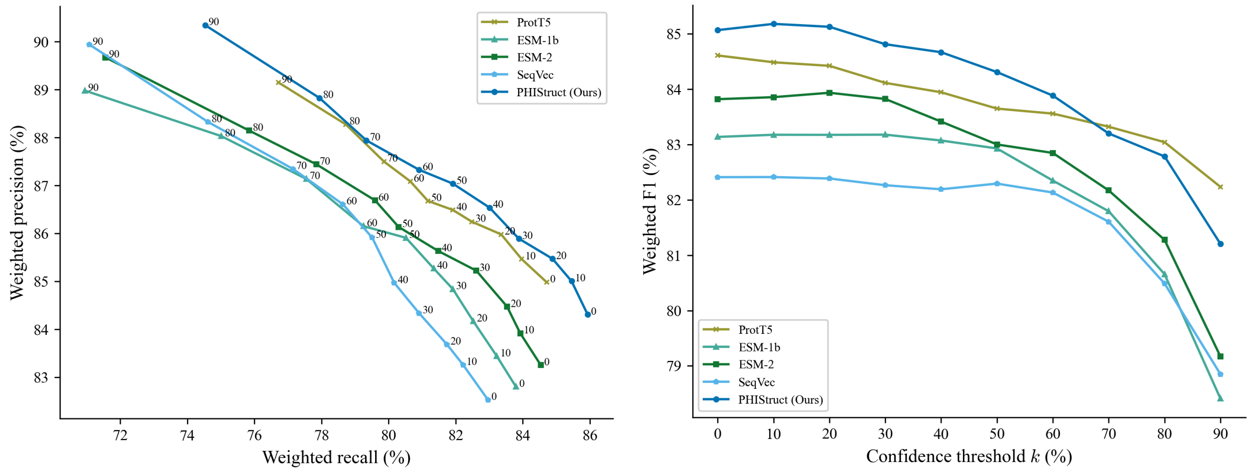

(a) (b)

Supplementary Fig. 25: Comparison of the performance of PHIStruct with same-architecture multilayer perceptron models that take in sequence-only embeddings. The maximum train-versus-test sequence similarity is set to $s = 100\%$. Performance is measured in terms of weighted metrics. (a) Precision-recall curves. The label of each point denotes the confidence threshold k (%) at which the performance was measured. (b) F1 scores. Higher values of k prioritize precision over recall, whereas lower values prioritize recall.

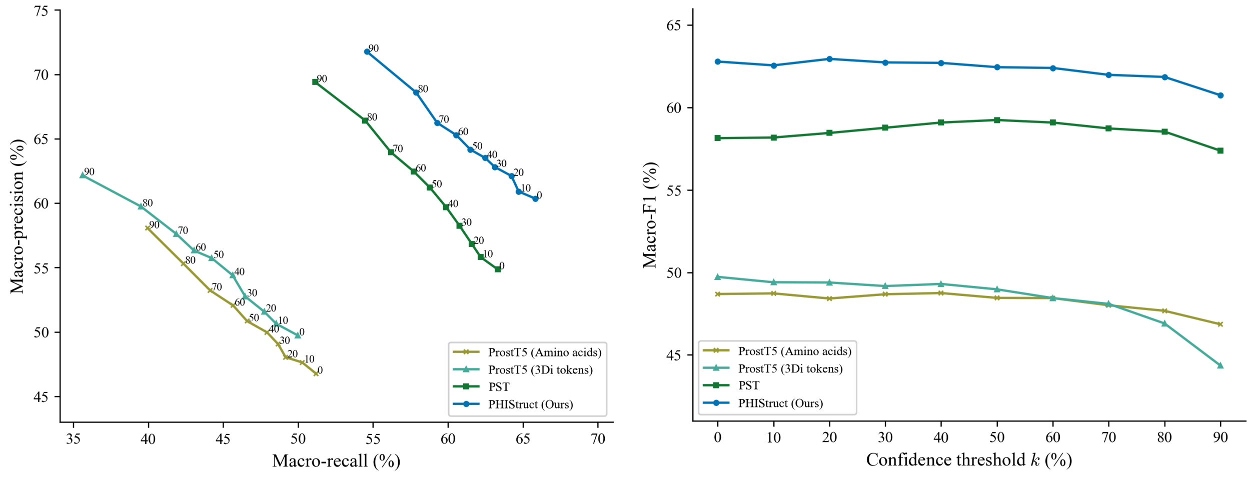

(a) (b)

Supplementary Fig. 26: Comparison of the performance of PHIStruct with same-architecture multilayer perceptron models that take in structure-aware protein embeddings other than SaProt. The maximum train-versus-test sequence similarity is set to $s = 60\%$. Performance is measured in terms of class-averaged (macro) metrics. (a) Precision-recall curves. The label of each point denotes the confidence threshold k (%) at which the performance was measured. (b) F1 scores. Higher values of k prioritize precision over recall, whereas lower values prioritize recall.

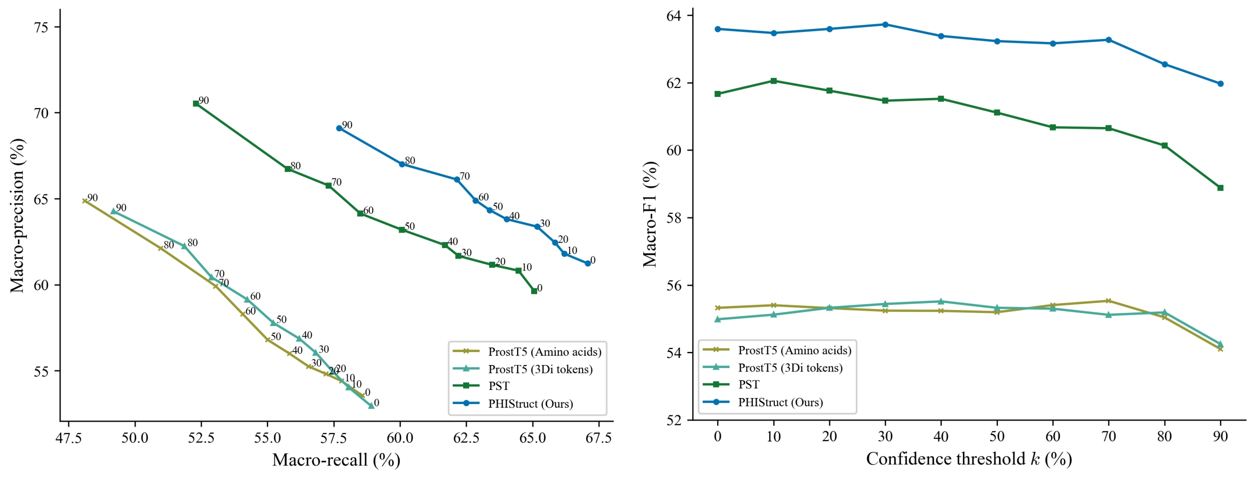

(a) (b)

Supplementary Fig. 27: Comparison of the performance of PHIStruct with same-architecture multilayer perceptron models that take in structure-aware protein embeddings other than SaProt. The maximum train-versus-test sequence similarity is set to $s = 80\%$. Performance is measured in terms of class-averaged (macro) metrics. (a) Precision-recall curves. The label of each point denotes the confidence threshold k (%) at which the performance was measured. (b) F1 scores. Higher values of k prioritize precision over recall, whereas lower values prioritize recall.

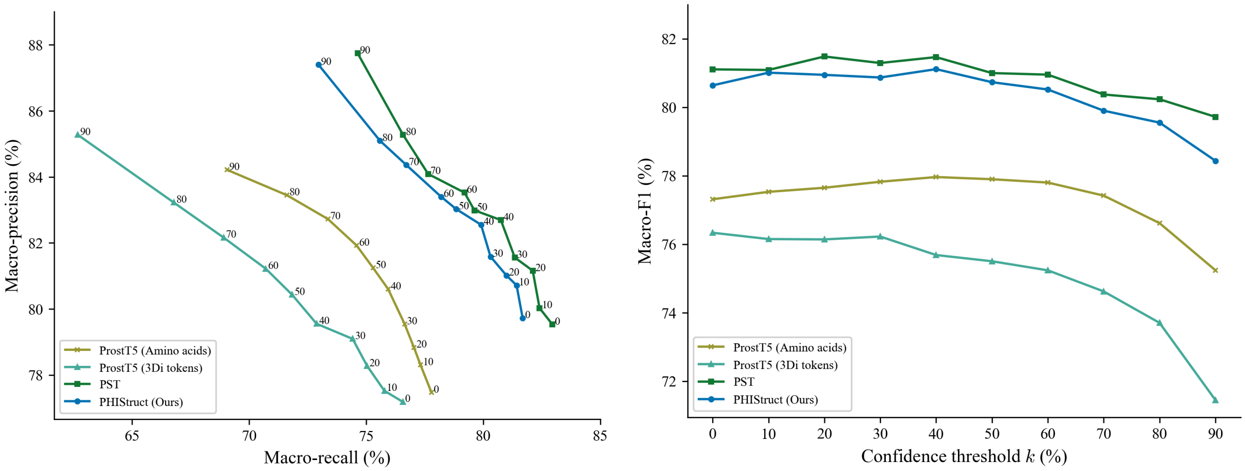

(a) (b)

Supplementary Fig. 28: Comparison of the performance of PHIStruct with same-architecture multilayer perceptron models that take in structure-aware protein embeddings other than SaProt. The maximum train-versus-test sequence similarity is set to $s = 100\%$. Performance is measured in terms of class-averaged (macro) metrics. (a) Precision-recall curves. The label of each point denotes the confidence threshold k (%) at which the performance was measured. (b) F1 scores. Higher values of k prioritize precision over recall, whereas lower values prioritize recall.

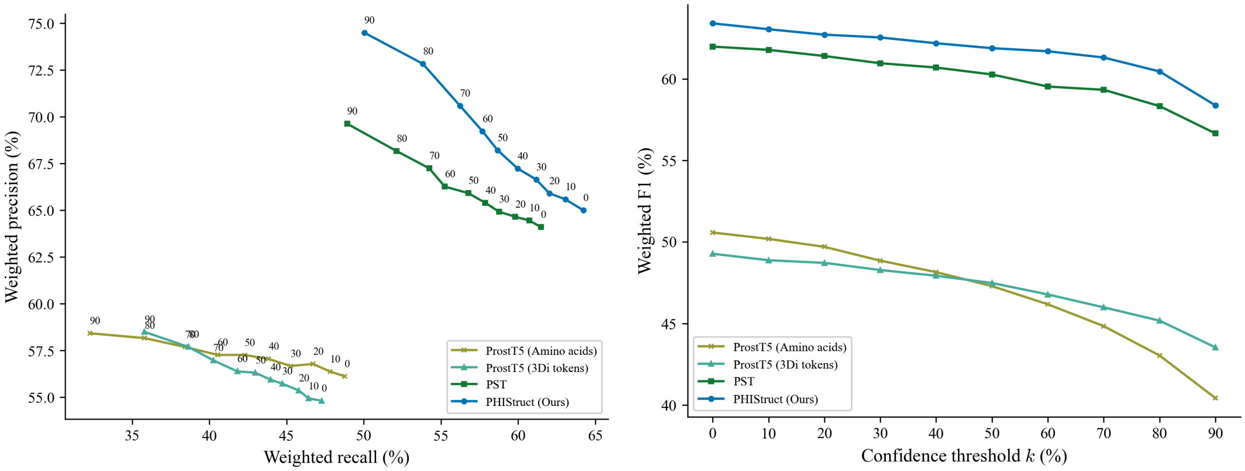

(a) (b)

Supplementary Fig. 29: Comparison of the performance of PHIStruct with same-architecture multilayer perceptron models that take in structure-aware protein embeddings other than SaProt. The maximum train-versus-test sequence similarity is set to $s = 40\%$. Performance is measured in terms of weighted metrics. (a) Precision-recall curves. The label of each point denotes the confidence threshold k (%) at which the performance was measured. (b) F1 scores. Higher values of k prioritize precision over recall, whereas lower values prioritize recall.

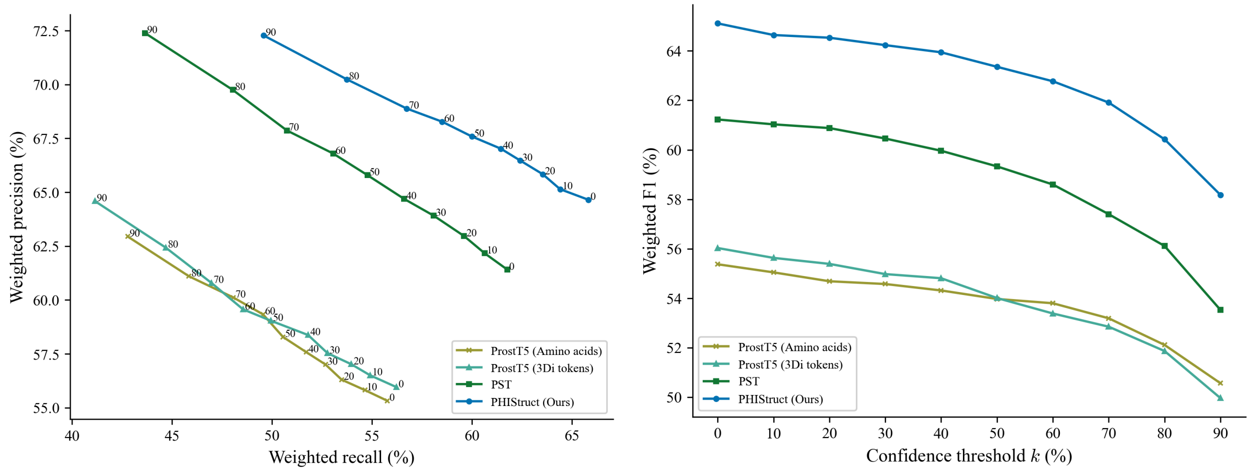

(a) (b)

Supplementary Fig. 30: Comparison of the performance of PHIStruct with same-architecture multilayer perceptron models that take in structure-aware protein embeddings other than SaProt. The maximum train-versus-test sequence similarity is set to $s = 60\%$. Performance is measured in terms of weighted metrics. (a) Precision-recall curves. The label of each point denotes the confidence threshold k (%) at which the performance was measured. (b) F1 scores. Higher values of k prioritize precision over recall, whereas lower values prioritize recall.

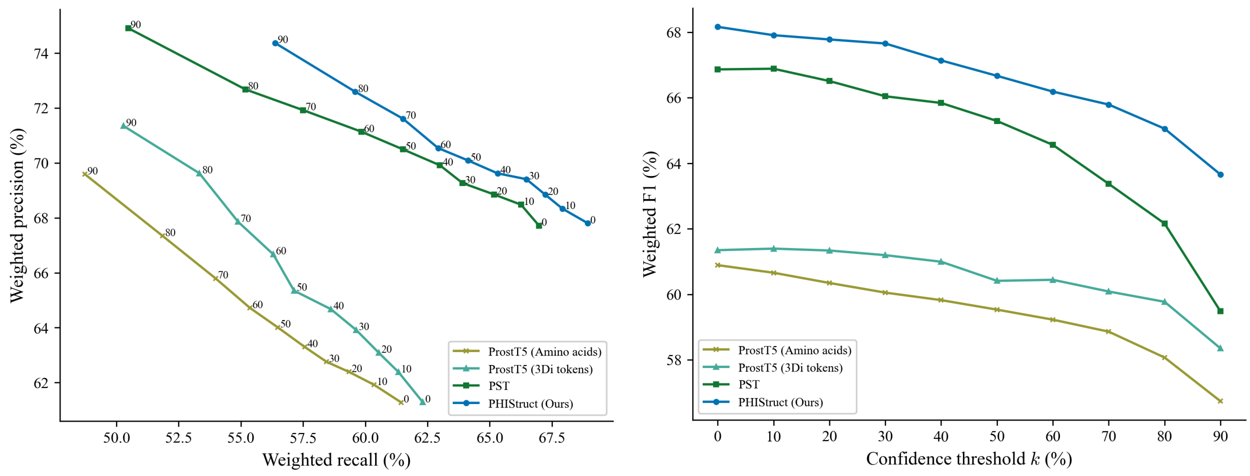

(a) (b)

Supplementary Fig. 31: Comparison of the performance of PHIStruct with same-architecture multilayer perceptron models that take in structure-aware protein embeddings other than SaProt. The maximum train-versus-test sequence similarity is set to $s = 80\%$. Performance is measured in terms of weighted metrics. (a) Precision-recall curves. The label of each point denotes the confidence threshold k (%) at which the performance was measured. (b) F1 scores. Higher values of k prioritize precision over recall, whereas lower values prioritize recall.

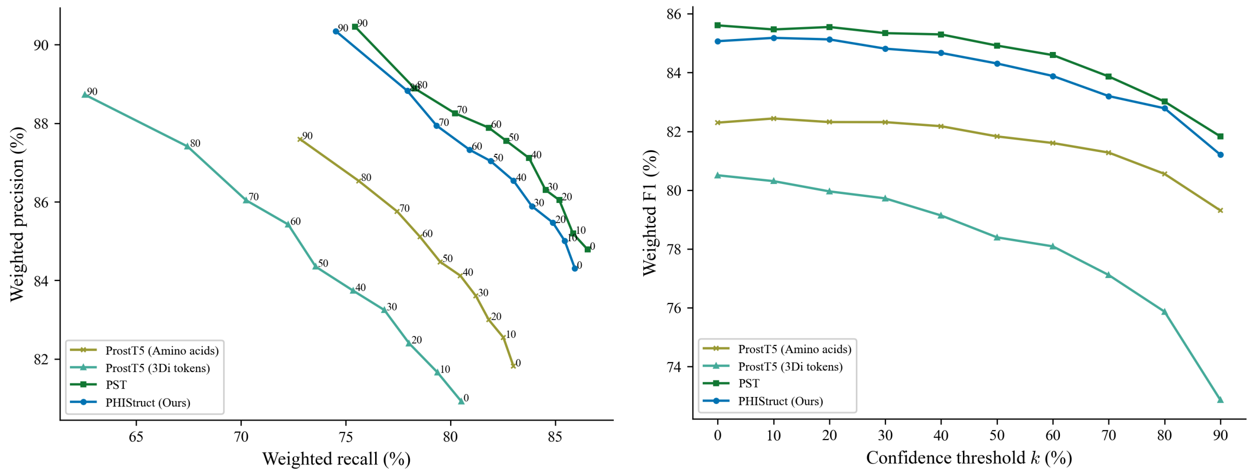

(a) (b)

Supplementary Fig. 32: Comparison of the performance of PHIStruct with same-architecture multilayer perceptron models that take in structure-aware protein embeddings other than SaProt. The maximum train-versus-test sequence similarity is set to $s = 100\%$. Performance is measured in terms of weighted metrics. (a) Precision-recall curves. The label of each point denotes the confidence threshold k (%) at which the performance was measured. (b) F1 scores. Higher values of k prioritize precision over recall, whereas lower values prioritize recall.

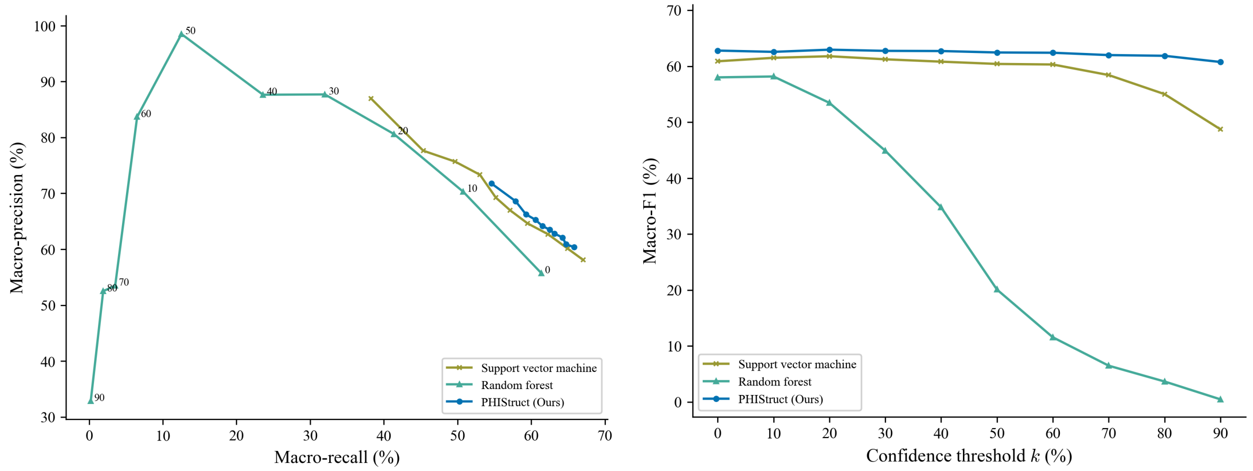

(a) (b)

Supplementary Fig. 33: Comparison of the performance of PHIStruct with other downstream classifiers take in the same SaProt embeddings. The maximum train-versus-test sequence similarity is set to $s = 60\%$. Performance is measured in terms of class-averaged (macro) metrics. (a) Precision-recall curves. The label of each point denotes the confidence threshold k (%) at which the performance was measured. (b) F1 scores. Higher values of k prioritize precision over recall, whereas lower values prioritize recall.

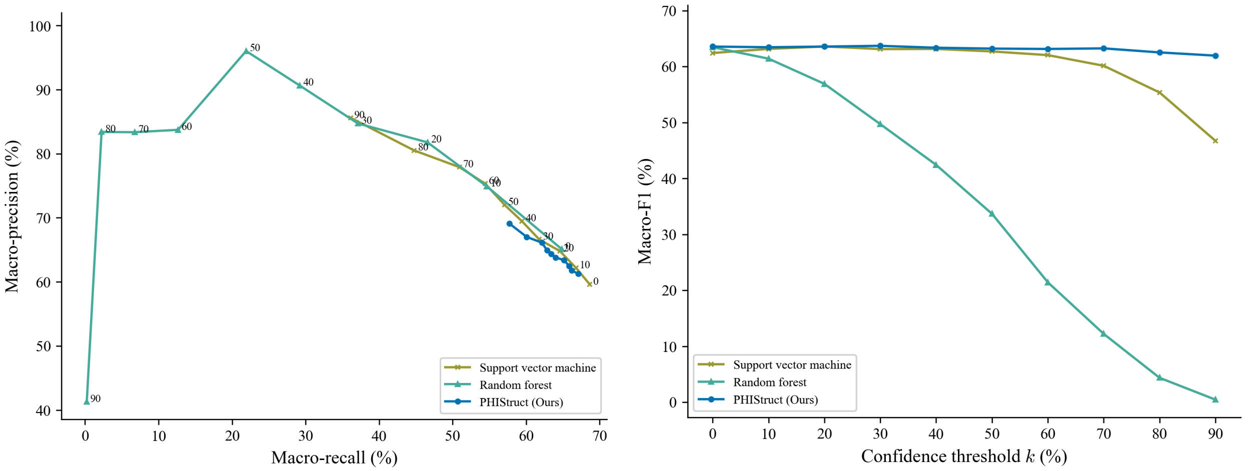

(a) (b)

Supplementary Fig. 34: Comparison of the performance of PHIStruct with other downstream classifiers take in the same SaProt embeddings. The maximum train-versus-test sequence similarity is set to $s = 80\%$. Performance is measured in terms of class-averaged (macro) metrics. (a) Precision-recall curves. The label of each point denotes the confidence threshold k (%) at which the performance was measured. (b) F1 scores. Higher values of k prioritize precision over recall, whereas lower values prioritize recall.

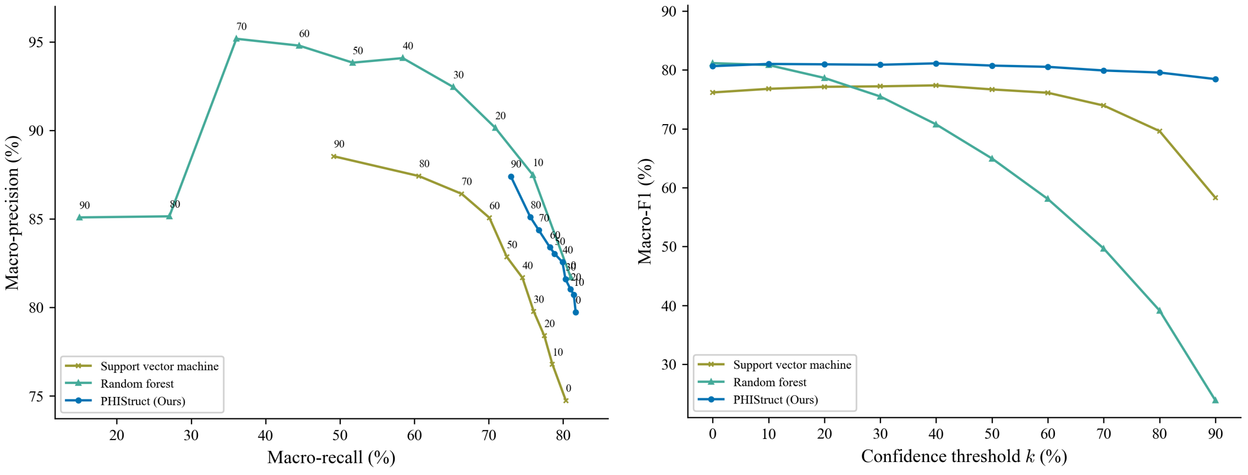

(a) (b)

Supplementary Fig. 35: Comparison of the performance of PHIStruct with other downstream classifiers take in the same SaProt embeddings. The maximum train-versus-test sequence similarity is set to $s = 100\%$. Performance is measured in terms of class-averaged (macro) metrics. (a) Precision-recall curves. The label of each point denotes the confidence threshold k (%) at which the performance was measured. (b) F1 scores. Higher values of k prioritize precision over recall, whereas lower values prioritize recall.

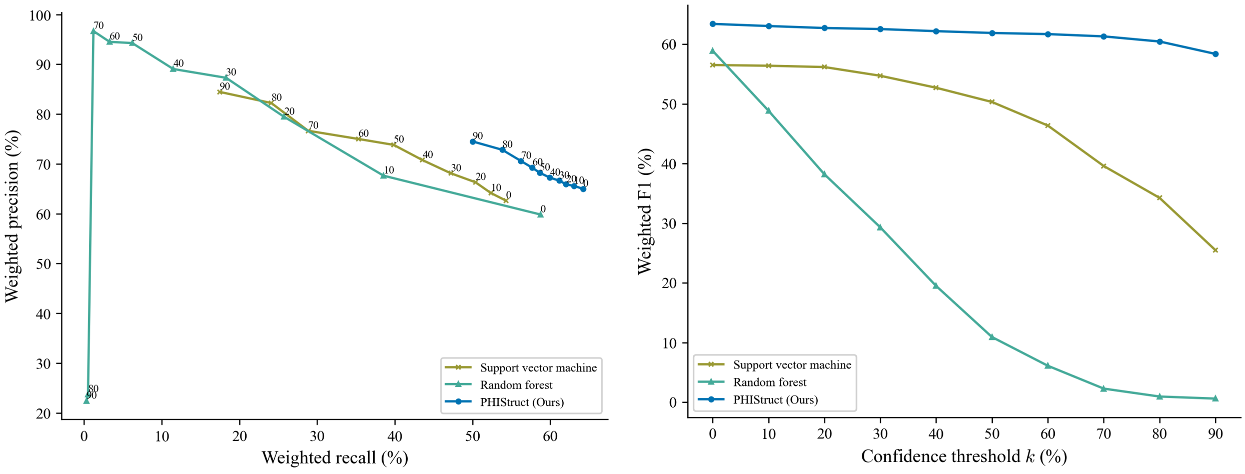

(a) (b)

Supplementary Fig. 36: Comparison of the performance of PHIStruct with other downstream classifiers take in the same SaProt embeddings. The maximum train-versus-test sequence similarity is set to $s = 40\%$. Performance is measured in terms of weighted metrics. (a) Precision-recall curves. The label of each point denotes the confidence threshold k (%) at which the performance was measured. (b) F1 scores. Higher values of k prioritize precision over recall, whereas lower values prioritize recall.

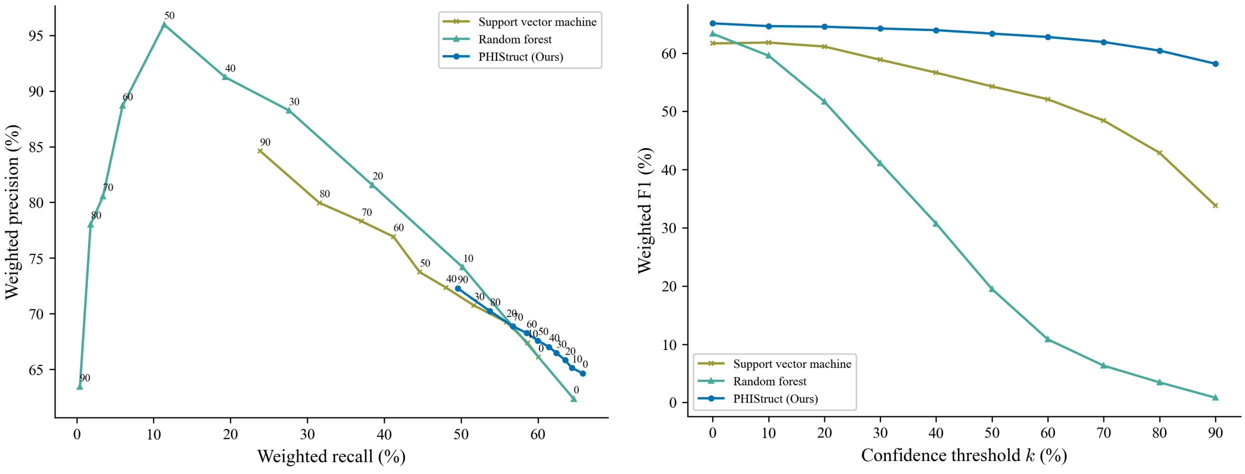

(a) (b)

Supplementary Fig. 37: Comparison of the performance of PHIStruct with other downstream classifiers take in the same SaProt embeddings. The maximum train-versus-test sequence similarity is set to $s = 60\%$. Performance is measured in terms of weighted metrics. (a) Precision-recall curves. The label of each point denotes the confidence threshold k (%) at which the performance was measured. (b) F1 scores. Higher values of k prioritize precision over recall, whereas lower values prioritize recall.

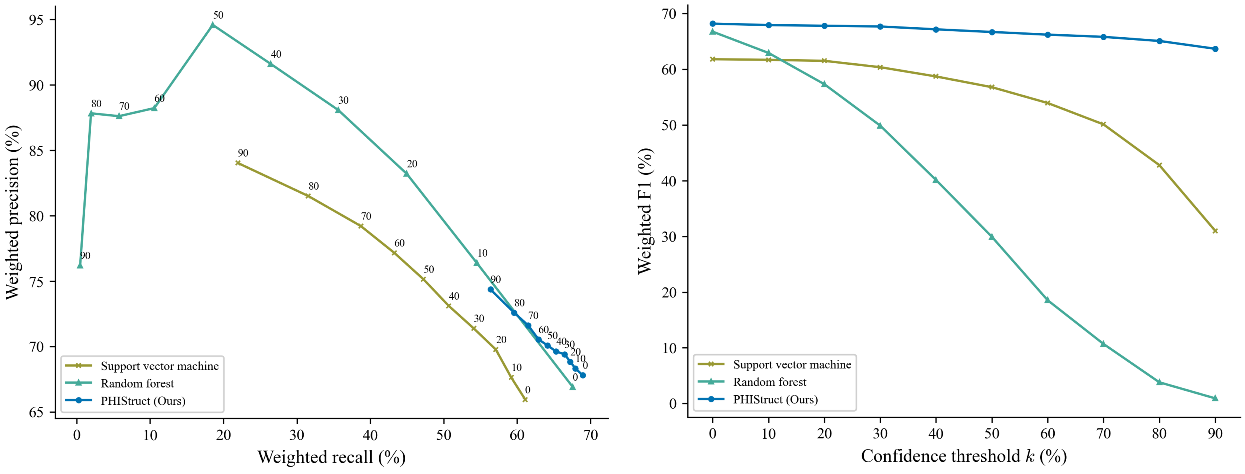

(a) (b)

Supplementary Fig. 38: Comparison of the performance of PHIStruct with other downstream classifiers take in the same SaProt embeddings. The maximum train-versus-test sequence similarity is set to $s = 80\%$. Performance is measured in terms of weighted metrics. (a) Precision-recall curves. The label of each point denotes the confidence threshold k (%) at which the performance was measured. (b) F1 scores. Higher values of k prioritize precision over recall, whereas lower values prioritize recall.

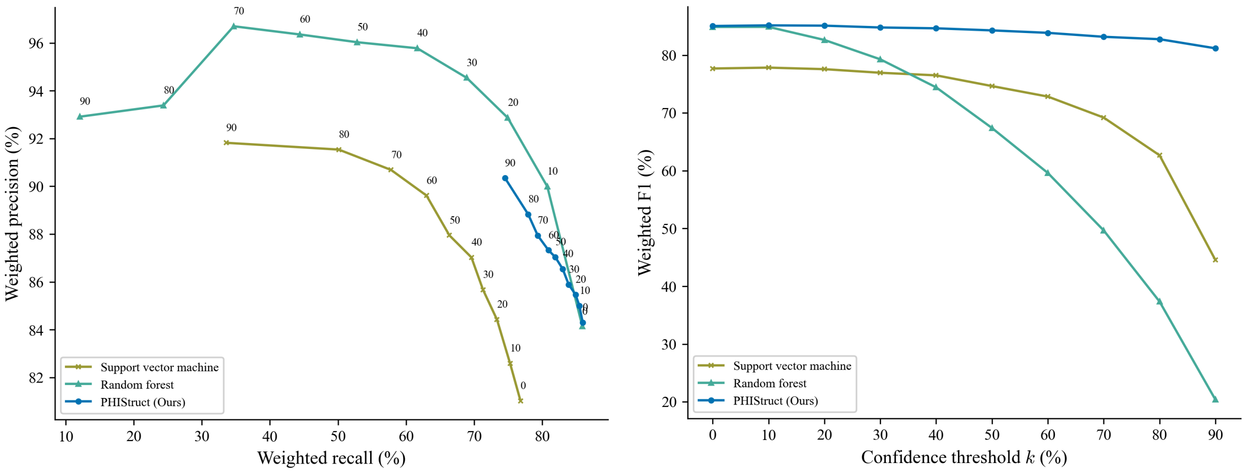

(a) (b)

Supplementary Fig. 39: Comparison of the performance of PHIStruct with other downstream classifiers take in the same SaProt embeddings. The maximum train-versus-test sequence similarity is set to $s = 100\%$. Performance is measured in terms of weighted metrics. (a) Precision-recall curves. The label of each point denotes the confidence threshold k (%) at which the performance was measured. (b) F1 scores. Higher values of k prioritize precision over recall, whereas lower values prioritize recall.

Supplementary Table 9: Protein language models used in benchmarking PHIStruct's performance

Model	Architecture	Number of parameters	Pretraining dataset
Sequence-only protein language models			
ProtT5 (Elnaggar et al. 2022)	Transformer	3 billion (ProtT5-XL)	BFD (Steinegger et al. 2019, Steinegger and Söding 2018), fine-tuned on UniRef-50 (Suzek et al. 2007)
ESM-1b (Rives et al. 2021)	Transformer	650 million	UniRef-50 (Suzek et al. 2007)
ESM-2 (Lin et al. 2023)	Transformer	650 million	UniRef-50 (Suzek et al. 2007)
Structure-aware protein language models			
ProstT5 (Heinzinger et al. 2024)	Transformer (based on the 3-billion-parameter version of ProtT5)	3 billion	AlphaFold DB (Varadi et al. 2021)
PST (Chen et al. 2024)	Graph isomorphism network and transformer (based on the 650-million-parameter version of ESM-2)	1 billion	AlphaFold Swiss-Prot (Varadi et al. 2021)
